# SIRT6 enhances oxidative phosphorylation in breast cancer and promotes mammary tumorigenesis in mice

**DOI:** 10.1186/s40170-021-00240-1

**Published:** 2021-01-22

**Authors:** Pamela Becherini, Irene Caffa, Francesco Piacente, Patrizia Damonte, Valerio G. Vellone, Mario Passalacqua, Andrea Benzi, Tommaso Bonfiglio, Daniele Reverberi, Amr Khalifa, Moustafa Ghanem, Ana Guijarro, Luca Tagliafico, Marzia Sucameli, Angelica Persia, Fiammetta Monacelli, Michele Cea, Santina Bruzzone, Silvia Ravera, Alessio Nencioni

**Affiliations:** 1 Department of Internal Medicine and Medical Specialties (DIMI), University of Genoa, V.le Benedetto XV 6, 16132 Genoa, Italy; 2 Ospedale Policlinico San Martino IRCCS, Largo Rosanna Benzi 10, 16132 Genoa, Italy; 3 Department of Experimental Medicine (DIMES), University of Genoa, V.le Benedetto XV 1, 16132 Genoa, Italy; 4 Department of Integrated, Surgical and Diagnostic Sciences (DISC), University of Genoa, L.go Rosanna Benzi 8, 16132 Genoa, Italy

**Keywords:** SIRT6, Breast cancer, Cancer metabolism, Mammary tumorigenesis, Oxidative phosphorylation

## Abstract

**Background:**

Sirtuin 6 (SIRT6) is a NAD^+^-dependent deacetylase with key roles in cell metabolism. High SIRT6 expression is associated with adverse prognosis in breast cancer (BC) patients. However, the mechanisms through which SIRT6 exerts its pro-oncogenic effects in BC remain unclear. Here, we sought to define the role of SIRT6 in BC cell metabolism and in mouse polyoma middle T antigen (PyMT)-driven mammary tumors.

**Methods:**

We evaluated the effect of a heterozygous deletion of *Sirt6* on tumor latency and survival of mouse mammary tumor virus (MMTV)-PyMT mice. The effect of SIRT6 silencing on human BC cell growth was assessed in MDA-MB-231 xenografts. We also analyzed the effect of *Sirt6* heterozygous deletion, of SIRT6 silencing, and of the overexpression of either wild-type (WT) or catalytically inactive (H133Y) SIRT6 on BC cell pyruvate dehydrogenase (PDH) expression and activity and oxidative phosphorylation (OXPHOS), including respiratory complex activity, ATP/AMP ratio, AMPK activation, and intracellular calcium concentration.

**Results:**

The heterozygous *Sirt6* deletion extended tumor latency and mouse survival in the MMTV-PyMT mouse BC model, while SIRT6 silencing slowed the growth of MDA-MB-231 BC cell xenografts. WT, but not catalytically inactive, SIRT6 enhanced PDH expression and activity, OXPHOS, and ATP/AMP ratio in MDA-MB-231 and MCF7 BC cells. Opposite effects were obtained by SIRT6 silencing, which also blunted the expression of genes encoding for respiratory chain proteins, such as *UQCRFS1*, *COX5B*, *NDUFB8*, and *UQCRC2*, and increased AMPK activation in BC cells. In addition, SIRT6 overexpression increased, while SIRT6 silencing reduced, intracellular calcium concentration in MDA-MB-231 cells. Consistent with these findings, the heterozygous *Sirt6* deletion reduced the expression of OXPHOS-related genes, the activity of respiratory complexes, and the ATP/AMP ratio in tumors isolated from MMTV-PyMT mice.

**Conclusions:**

Via its enzymatic activity, SIRT6 enhances PDH expression and activity, OXPHOS, ATP/AMP ratio, and intracellular calcium concentration, while reducing AMPK activation, in BC cells. Thus, overall, SIRT6 inhibition appears as a viable strategy for preventing or treating BC.

**Supplementary Information:**

The online version contains supplementary material available at 10.1186/s40170-021-00240-1.

## Background

Breast cancer (BC) is the most frequently diagnosed malignancy, with 1.7 million new diagnoses/year (23% of cancer diagnoses) [1]. In 2018, there were more than six hundred thousand BC-related deaths globally, and it is estimated that this death toll will rise to one million deaths/year by 2040 without major advances in BC prevention or treatment [1]. Thus, there remains a crucial need to enhance our understanding of BC biology and to define new viable therapeutic targets for this condition.

Neoplastic transformation involves a metabolic reprogramming to support the biosynthesis of macromolecules that are needed for cell division and growth [2]. Such changes are similar to those occurring in highly proliferative normal cells during embryogenesis, wound healing, and immune response [3]. Cancer cells acquire mutations in oncogenes and in tumor suppressors that enhance glycolysis and anabolic metabolism in the absence of external signals (Warburg effect) [4]. In addition, in contrast to Warburg’s initial hypothesis, mitochondrial oxidative phosphorylation (OXPHOS) is also highly active and actually plays a key role in cancer cells. Specifically, while glycolysis represents a source of molecules necessary for biomass enhancement, OXPHOS appears to underlie anabolic metabolism, cell proliferation, cancer stemness, and metastasis [3].

Sirtuin 6 (SIRT6) is a member of the sirtuin family of NAD^+^-dependent deacetylases. In mammals, seven sirtuin isoforms exist (SIRT1-7), with different subcellular localization, catalytic activity, targets, and functions. SIRT6 is a multifunctional nuclear protein, involved in different physiological processes and pathological conditions, such as genome stability, longevity, glucose metabolism, neurodegenerative, heart and liver diseases, diabetes, inflammation, cancer, and bone disorders [5–8]. SIRT6 role in cancer was proposed to be tissue-dependent [9, 10]. In gastrointestinal tumors, SIRT6 was reported to have tumor-suppressive effects thanks to its ability to prevent HIF-1α and NF-κB activity [11]. However, in other types of malignancies, such as multiple myeloma, acute myeloid leukemia, and non-melanoma skin cancer, and in pancreatic cancer cells, SIRT6 was reported to have tumor-promoting effects thanks to its ability to enhance tumor DNA repair, secretion of pro-inflammatory and pro-angiogenic factors, Ca^2+^ signaling, cell migration, and cancer cell de-differentiation [12–15]. In BC patients, high tumor levels of SIRT6 were shown to enhance BC cell resistance to chemotherapeutics and to promote BC survival, migration, and invasion through the expression of cyclin D1, NF-κB, β-catenin, and matrix metalloproteinase 9 (MMP9) [16, 17].

Here, we focused on the effect of SIRT6 depletion on mammary tumorigenesis using the mammary tumor virus promoter (MMTV)-polyomavirus middle T antigen (PyMT) mouse BC model as well as MDA-MB-231 xenografts and assessed the role of SIRT6 in BC mitochondrial metabolism.

## Methods

### Mice

Six- to 8-week-old female BALB/c athymic nude mice (Hsd:Athymic Nude-*Foxn1*^*nu*^) were purchased from Envigo (Italy) and used to generate a xenograft mouse BC model (see the “Tumor models” section). Mice were housed in temperature- and light-controlled conditions (12-h light cycle) with food and water ad libitum. Mice were acclimatized for 2 weeks after their arrival. 129SvJ female mice heterozygous for *Sirt6* (*Sirt6*^*+/−*^) were a kind gift from Dr. Raul Mostoslavsky (MGH Cancer Center, Boston, MA, USA) [18]; they were used to generate a transgenic model of spontaneous BC (see the “Tumor models” section). Male mice heterozygous for *MMTV-PyMT* (*MMTV-PyMT*^*+/−*^) in a 129/Ola genetic background were provided by Dr. Thorsten Berger (The Campbell Family Institute for Breast Cancer Research Ontario Cancer Institute) [19]. The MMTV-PyMT colony was maintained through heterozygous males because the heterozygous females develop tumors early and are therefore unable to breastfeed any puppies. Three- to 9-month-old *Sirt6*^*+/−*^ female mice were bred with *MMTV-PyMT*^*+/−*^ male mice to generate two different groups of interest: *MMTV-PyMT*^*+/−*^*; Sirt6*^*+/+*^ control mice and *MMTV-PyMT*^*+/−*^*; Sirt6*^*+/−*^ experimental mice. The mammary glands were isolated from 12-week-old *Sirt6*^*+/+*^ and *Sirt6*^*+/−*^ mice by standard procedures, flash-frozen, and stored at − 80 °C until utilization for RNA isolation, ATP, and AMP measurements and respiratory complex activity assays. All animal experiments were performed in accordance with the relevant guidelines and regulations (Italian 26/2014 and EU 2010/63/UE directives) and were approved by the Licensing and Ethical Committee (OPBA) of Ospedale Policlinico San Martino IRCCS and by the Italian Ministry of Health.

### Cell lines

MCF7, MDA-MB-231, and Phoenix cell lines were purchased from ATCC and were cultured in RPMI 1640 medium supplemented with 10% heat-inactivated fetal bovine serum and penicillin-streptomycin (50 U/mL) (LifeTechnologies, Italy).

### Viral production and transduction

The retroviral plasmids for silencing SIRT6 (short hairpin#2 - sh2) and for overexpressing wild-type (WT) or catalytically inactive (H133Y) SIRT6, and the respective vectors (PRS for the shRNA and pBABEpuro for the expression plasmids), were described previously [20, 21]. For retroviral transductions, 1 × 10^6^ Phoenix cells were plated in 4 ml medium in 6-cm dishes and allowed to adhere for 24 h. Thereafter, cells were transfected with 4 μg of plasmid DNA using TransIT-293 (Mirus Bio, Madison, WI, USA) according to the manufacturer’s instructions. Viral supernatants were harvested after 36, 48, 60, and 72 h and used to infect MCF7 cells (5 × 10^5^) and MDA-MB-231 (3 × 10^5^) cells in 10-cm dishes in the presence of 5 μg/ml protamine sulfate. Successfully infected cells were selected using 1.5 μg/ml puromycin (MCF7) or 1 μg/ml puromycin (MDA-MB-231).

### Western blot analysis

After transduction, 6 × 10^5^ MCF7 and 4 × 10^5^ MDA-MB-231 cells were plated in 10-cm dishes and allowed to adhere for 48 h. Thereafter, cells were lysed in ice-cold lysis buffer [50 mM Tris-HCl (pH 7.5), 150 mM NaCl, 1% Nonidet P-40, Protease Inhibitor Cocktail, and Phosphatase Inhibitor Cocktail 2 from Sigma Aldrich], and the protein concentration was determined according to a standard Bradford assay. Proteins (35 μg) were separated by SDS-PAGE and transferred to a PVDF membrane (Immobilon-P, Millipore, Vimodrone, Italy). Proteins of interest were detected with the following antibodies: anti-SIRT6 (1:1000, #2590 Cell Signaling), anti-Phospho-AMPK (1:1000, PA5-17831 Thermo Scientific), anti-AMPK (1:1000, PA5-29679 Thermo Scientific), anti-pyruvate dehydrogenase (1:1000, #3205 Cell Signaling), anti-GAPDH (1:1000, #5174 Cell Signaling), anti-Vinculin H-300 (1:200, sc-5573 Santa Cruz Biotechnology), anti-SIRT6 (1:1000, LS-C49020 LifeSpan BioSciences; for detecting mouse Sirt6), and anti-αtubulin (1:1000, DM1A: sc-32293, Santa Cruz Biotechnology). Band intensities were quantified by the Quantity One SW software (Bio-Rad Laboratories, Inc.) using standard ECL.

Tumor masses were excised from xenograft mice and homogenized in ice-cold lysis buffer with an electric homogenizer. The samples were maintained in constant agitation for 2 h at 4 °C, on an orbital shaker in a cold room. Finally, the samples were centrifuged for 20 min at 12,000 rpm at 4 °C in a microcentrifuge, the supernatants were collected, and the protein content was evaluated by Bradford assay.

### Quantitative real-time PCR

RNA extraction, cDNA synthesis, and quantitative real-time polymerase chain reaction (QPCR) were performed as described elsewhere [22]. Gene-specific primers were purchased from Sigma-Aldrich (Italy) or Thermo Fisher (Italy) and are listed in Supplementary Table 1. Comparisons in the gene expression were performed using the 2^−ΔΔCt^ method. β-Actin was used as the housekeeping gene.

### Mitochondrial mass determination

For mitochondrial mass determination, 2 × 10^5^ MDA-MB-231 cells were stained with 200 nM Mitotracker deep red (M22426, Thermo Fisher, Italy) according to the manufacturer’s instructions and immediately analyzed by flow cytometry (FACS Aria, Becton Dickinson, Germany) by acquiring 10,000 events. The Mitotracker deep red mean fluorescence intensity (MFI) was also estimated. Changes in mitochondrial mass as a consequence of either SIRT6 overexpression or silencing were also evaluated by estimating mitochondrial DNA (mtDNA) to nuclear DNA (nDNA) ratio. To this end, 10^6^ MDA-MB-231 cells were washed with PBS, centrifuged at 1.200 rpm for 5 min at 4 °C and resuspended in 100 μl lysis buffer (100 mM Tris-HCl pH 8, 200 mM NaCl, 5 mM EDTA, 0.2% SDS) with freshly added proteinase K (0.2 mg/ml). After 2 h incubation at 56 °C, samples were processed as described in "DNA extraction and mouse genotyping" (see below). One hundred nanograms of DNA was used to perform QPCR with SYBR green-based detection. Relative mtDNA:nDNA ratio was calculated using the 2^−ΔΔCt^ method by targeting the nuclear-encoded gene, human B2M, and the mitochondrial-encoded gene, human tRNALeu. Gene-specific primers are listed in Supplementary Table 1.

### Analysis of mitochondrial morphology by Mitotracker deep red

The analysis of mitochondrial morphological features was performed as described elsewhere [23]. In brief, 4 × 10^4^ MDA-MB-231 cells were plated on glass coverslips (Thermo Scientific Nunc Lab-Tek II chamber slide system, Italy) and allowed to adhere overnight. Thereafter, cells were stained with Mitotracker deep red (Thermo Fisher, Italy) according to the manufacturer’s instructions and analyzed by confocal microscopy. Images were collected using a three-channel TCS SP2 laser-scanning confocal microscope (Leica Microsystems, Wetzlar, Germany). Mitochondrial solidity was defined as the fraction of pixels contained with a convex polygon (fitted around a mitochondrion) that is also mitochondrial pixels. Low solidity (close to 0.0) tends to describe highly tortuous mitochondria that are not uniform in shape, while high solidity values (close to 1.0) tend to describe mitochondria that are more uniform in shape and do not contain a high level of branching. Mitochondrial perimeter was defined as the number of exterior mitochondrial pixels multiplied by the length of the pixels, in microns. Circularity was calculated by the following formula: circularity = 4*π* × (area/perimeter^2^). Mitochondria exhibiting a perfect circular shape have a circularity value close to 1.0, whereas more elongated mitochondria have a circularity value that is close to 0.0.

### Tumor models

To generate the MDA-MB-231 BC xenograft model, 2 × 10^6^ MDA-MB-231 cells engineered with the PRS vector (VECTOR) or with a SIRT6-targeting shRNA (SIRT6-sh) were subcutaneously injected into both flanks of female BALB/c athymic nude mice. Tumor growth was monitored over time measuring tumor sizes with a manual caliper, and the tumor volume was registered twice a week. Mice were sacrificed when the tumor reached a volume of about 1.5 cm^3^ [the maximal tumor volume permitted by our Institutional Animal Care and Use Committee (IACUC)]. A genetically modified model that spontaneously develops breast tumors was also used (see above). *MMTV-PyMT*^*+/−*^; *Sirt6*^*+/+*^ and *MMTV-PyMT*^*+/−*^; *Sirt6*^*+/−*^ mice were monitored palpating the mammary glands: when the tumors appeared as palpable masses, the age of the mice was recorded to study tumor latency and mouse survival. Tumor volume was calculated using the following formula: tumor volume = (*w*^2^ × *W*) × *π*/6, where *w* and *W* are “minor side” and “major side” (in mm), respectively. Blood glucose was measured in blood samples that were obtained from the tail of 3-month-old *Sirt6*^*+/+*^, *Sirt6*^*+/−*^, *MMTV-PyMT*^*+/−*^*; Sirt6*^*+/+*^, and *MMTV-PyMT*^*+/−*^*; Sirt6*^*+/−*^ mice using Glucomen Areo 2k (Menarini, Italy). Organs [heart, lung, kidney, spleen, and mesenteric visceral adipose tissue (VAT)] from 4-week-old *MMTV-PyMT*^*+/−*^*; Sirt6*^*+/+*^ and *MMTV-PyMT*^*+/−*^*; Sirt6*^*+/−*^ mice were collected by standard procedures, flash-frozen, and stored at − 80 °C until utilization for RNA isolation.

### DNA extraction and mouse genotyping

DNA extraction from mouse tails was performed according to the following protocol: approximately 4 mm mouse tail was digested with 500 μl tail lysis buffer [5 mM EDTA, 0.2% sodium dodecyl sulfate (SDS), 200 mM NaCl, in 100 mM Tris-HCl, pH 8.0] containing 200 μg/ml proteinase K, in a 1.5-ml tube at 56 °C overnight with agitation (1,000 rpm on a heated shaker). The day after, samples were centrifuged at 14,000 rpm for 10 min at room temperature for debris removal. The supernatant was transferred to a clean tube with 500 μl isopropanol and inverted until DNA precipitation was observed. Subsequently, samples were centrifuged at 14,000 rpm for 5 min at RT, the supernatant was discarded, and the DNA pellet was washed by adding 300 μl of 70% ethanol and centrifuged at 14,000 rpm for 5 min at RT. After centrifugation, the ethanol solution was removed, and the DNA pellet was dried at RT or in a desiccator. Finally, the DNA pellet was resuspended in 35–100 μl DNase-free water. Genotyping was performed using Multiplex PCR Master Mix 2x (BR0200801, Biotechrabbit, Germany) to determine the presence of WT or KO allele of *Sirt6* and of *PyMT* genes. For *Sirt6* gene, three oligonucleotides were used, two specific for WT or KO allele and one in common for the two different alleles (WT: TTTCGTATAAGTCCAAGCCC, KO: GCAATAGCATCACAAATTTCAC, COMMON: GGAAGGACCTGGACAAG). WT allele primer pair (WT and COMMON) amplifies a fragment of 422 bp spanning across exon 8, while KO allele primer pair (KO and COMMON) amplifies a fragment of 300 bp spanning across exon 8. For *PyMT* gene, two oligonucleotides were used (PyMT 1: GGAAGCAAGTACTTCACAAGG and PyMT 2: GGAAAGTCACTAGGAGCAGGG) to amplify a fragment of 600 bp. PCR products were analyzed by gel electrophoresis with 2% precast agarose gels (54813, 2% seakem gold agarose, Lonza, Italy) at 100 V. The amplification products were visualized with a ChemiDoc XRS (BioRad, Italy) instrument.

### Mammary tissue morphologic analysis

To prepare whole mounts, the fourth inguinal mammary gland was excised from 30-day-old female *MMTV-PyMT*^*+/−*^*; Sirt6*^*+/+*^ and *MMTV-PyMT*^*+/−*^*; Sirt6*^*+/−*^ mice and spread out on a pre-cleaned microscope slide. The gland was fixed in Carnoy’s solution (75% ethanol–25% glacial acetic acid) overnight at RT. The day after, the slides were hydrated in 70% ethanol and water and stained overnight with carmine alum (07070, STEMCELL Technologies, Italy). After staining, the slides were rinsed in water, dehydrated in increasing concentrations of ethanol, and cleared in histolemon (454915, Carlo Erba, Italy). Photographic images of the whole mounts were acquired by Nikon SMZ1270 microscope using the X-Entry software. Terminal end buds (TEB) were counted in the whole mammary gland.

### Histopathological and immunohistochemical analysis of mammary tumors

After being sacrificed by cervical dislocation, *MMTV-PyMT*^*+/−*^*; Sirt6*^*+/+*^ and *MMTV-PyMT*^*+/−*^*; Sirt6*^*+/−*^ mice were refrigerated and immediately sent to the pathological anatomy laboratory. Each animal was fixed by immersion in 10% buffered formalin for 12–18 h, after inoculation of the fixative intraperitoneally and intrathoracically. Subsequently, each animal was eviscerated en-block and the organs seriated with consecutive sections in the craniocaudal sense. Bilateral paramedian incisions along the breast lines were also performed to sample mammary tumors. All samples were routinely processed and paraffin-embedded to obtain 3-μm-thick histological sections stained with hematoxylin-eosin. Microvessel density was determined by CD34 staining (anti-CD34, clone QBEnd/10, Ventana) as described in [24].

### Cell homogenate preparation for ATP and AMP measurements and respiratory complex activity assays

To remove the growth medium, cells were centrifuged at 1000*g* for 2 min. The pellet was suspended in phosphate-buffered saline (PBS) buffer and sonicated on ice, 2 times for 10 s, with a 30-s break. The total protein concentration was estimated by the Bradford method [25].

### Evaluation of intracellular ATP and AMP content

Quantification of ATP and AMP was based on the enzyme coupling method [26]. Twenty micrograms of total proteins was used. Briefly, ATP was assayed spectrophotometrically at 340 nm, following NADP^+^ reduction, at 25 °C. The reaction mixture contained the following: 1 mM NADP^+^, 10 mM MgCl_2_, 5 mM glucose, and 100 mM Tris-HCl, pH 7.4, in 1 ml final volume. Samples were analyzed before and after the addition of purified hexokinase and glucose-6-phosphate dehydrogenase (4 μg; HK+G6PD, HKG6PDH-RO, Sigma-Aldrich, Italy). AMP was assayed spectrophotometrically at 340 nm, following NADH oxidation. The reaction mixture contained the following: 75 mM KCl, 5 mM MgCl_2_, 0.2 mM ATP, 0.5 mM phosphoenolpyruvate, 0.2 mM NADH, 10 IU adenylate kinase (AK, M3003, Sigma-Aldrich, Italy), 25 IU pyruvate kinase plus 15 IU lactate dehydrogenase (PK+LDH, Sigma-Aldrich, Italy), and 100 mM Tris-HCl pH 8.0.

### Oxymetric analysis

Oxygen consumption was measured with an amperometric oxygen electrode (Unisense) in a closed chamber, magnetically stirred, at 37 °C. For each assay, 2 × 10^5^ cells were used. After cell permeabilization with 0.03 mg/ml digitonin for 10 min, samples were suspended in 137 mM NaCl, 5 mM KH_2_PO_4_, 5 mM KCl, 0.5 mM EDTA, 3 mM MgCl_2_, and 25 mM Tris-HCl, pH 7.4. To activate the pathway composed of complexes I, III, and IV, 5 mM pyruvate and 2.5 mM malate were added. To activate the pathway composed of complexes II, III, and IV, 20 mM succinate was used [27].

### Evaluation of F_1_F_o_-ATP synthase activity

F_1_F_o_-ATP synthase (ATP synthase) activity was detected by measuring the ATP production by the highly sensitive luciferin/luciferase method. The assays were conducted at 37 °C, for 2 min, and data were collected every 30 s. Cells (1 × 10^5^) were added to the incubation medium (0.1 ml final volume), which contained 50 mM KCl, 1 mM EGTA, 2 mM EDTA, 5 mM KH_2_PO_4_, 2 mM MgCl_2_, 0.6 mM ouabain, 1 mM P^1^,P^5^-Di(adenosine-5′) pentaphosphate, 0.040 mg/ml ampicillin, 0.2 mM adenosine diphosphate (ADP), 10 mM Tris-HCl pH 7.4, and the metabolic substrates (5 mM pyruvate + 2.5 mM malate or 20 mM succinate). Cells were equilibrated for 10 min at 37 °C, and then ATP synthesis was induced by the addition of 0.2 mM ADP. ATP synthesis was measured using the luciferin/luciferase ATP bioluminescence assay kit CLSII (11699695001, Roche) and a Luminometer (GloMax® 20/20 Luminometer, Promega). ATP standard solutions in the concentration range 10^−10^–10^−7^ M were used for calibration [27].

### Respiratory complex activity assay

The activity of the redox complexes I, III, and IV was measured, in a double beam spectrophotometer (UNICAM UV2, Analytical S.n.c.), at 25 °C. For each assay, 50 μg total proteins were employed, and the reaction was followed for 5 min, collecting data every 1 min [28]. The enzymatic activity was expressed as mIU/mg total protein (nanomoles/min/mg protein). Complex I (NADH-ubiquinone oxidoreductase) was assayed following the reduction of ferrocyanide, in the presence of NADH, at 420 nm; the reaction mixture contained 30 mM NADH, 40 mM potassium ferrocyanide, and 40 μM antimycin A in 10 mM phosphate buffer pH 7.2. Complex III (cytochrome c reductase) activity was measured at 550 nm following the reduction of oxidized cytochrome c. The reaction mixture contained 10 mM phosphate buffer pH 7.2, 0.03% oxidized cytochrome c, and 0.5 mM KCN. Complex IV (cytochrome c oxidase) was assayed following the oxidation of ascorbate-reduced cytochrome c at 550 nm, in a solution containing 10 mM phosphate buffer pH 7.2, 0.03% reduced cytochrome c, and 40 μM antimycin A. In both assays, the cytochrome c extinction coefficient was considered 19.1 × 10^−3^ M^−1^ cm^−1^, at 550 nm.

### Evaluation of pyruvate dehydrogenase expression and activity

The expression and activity of pyruvate dehydrogenase (PDH) were performed using the “Pyruvate dehydrogenase (PDH) Combo (Activity + Profiling) Microplate Assay Kit” provided by Abcam (ab110671, Abcam), following the manufacturer’s instructions. For each sample, 4 × 10^6^ MDA-MB-231 cells were used.

### Quantification and statistical analysis

Statistical analyses were performed with the GraphPad Prism software version 5 (GraphPad Software, San Diego, USA). Data are shown as mean ± SD. All parameters were tested by two-tailed Student’s *t* test (in all in vitro experiments), by two-way ANOVA followed by Bonferroni post hoc test (in vivo xenografts experiments), or log rank test (in vivo survival and latency experiments). *P* values less than 0.05 were considered significant.

## Results

### SIRT6 downregulation slows BC progression in *MMTV-PyMT* mouse model

To define the role of SIRT6 in mammary carcinogenesis, we utilized transgenic mice expressing the *PyMT* under the *MMTV* promoter, which is an established animal model of human BC [29, 30]. Specifically, we crossed *MMTV-PyMT*^*+/−*^ male mice with *Sirt6*^*+/−*^ female animals and compared tumor latency and mouse survival between *MMTV-PyMT*^*+/−*^*; Sirt6*^*+/+*^ and *MMTV-PyMT*^*+/−*^*; Sirt6*^*+/−*^ mice. This approach was chosen since complete *Sirt6* knockout results in postnatal lethality (*Sirt6*^*−/−*^ mice are typically only viable until weaning) [18]. Thus, since the typical tumor latency in *MMTV-PyMT* 129 mice is between 10 and 12 weeks [19], the use of *Sirt6*^*−/−*^ mice would not have allowed us to assess the effect of *Sirt6* depletion on mammary tumor development. Conversely, a heterozygous *Sirt6* deletion (*Sirt6*^*+/−*^) was shown to be compatible with mouse survival [18] and yet to produce biologically relevant effects, including enhancing adhesion molecule expression and exacerbating atherosclerosis [31].

**Fig. 1:**
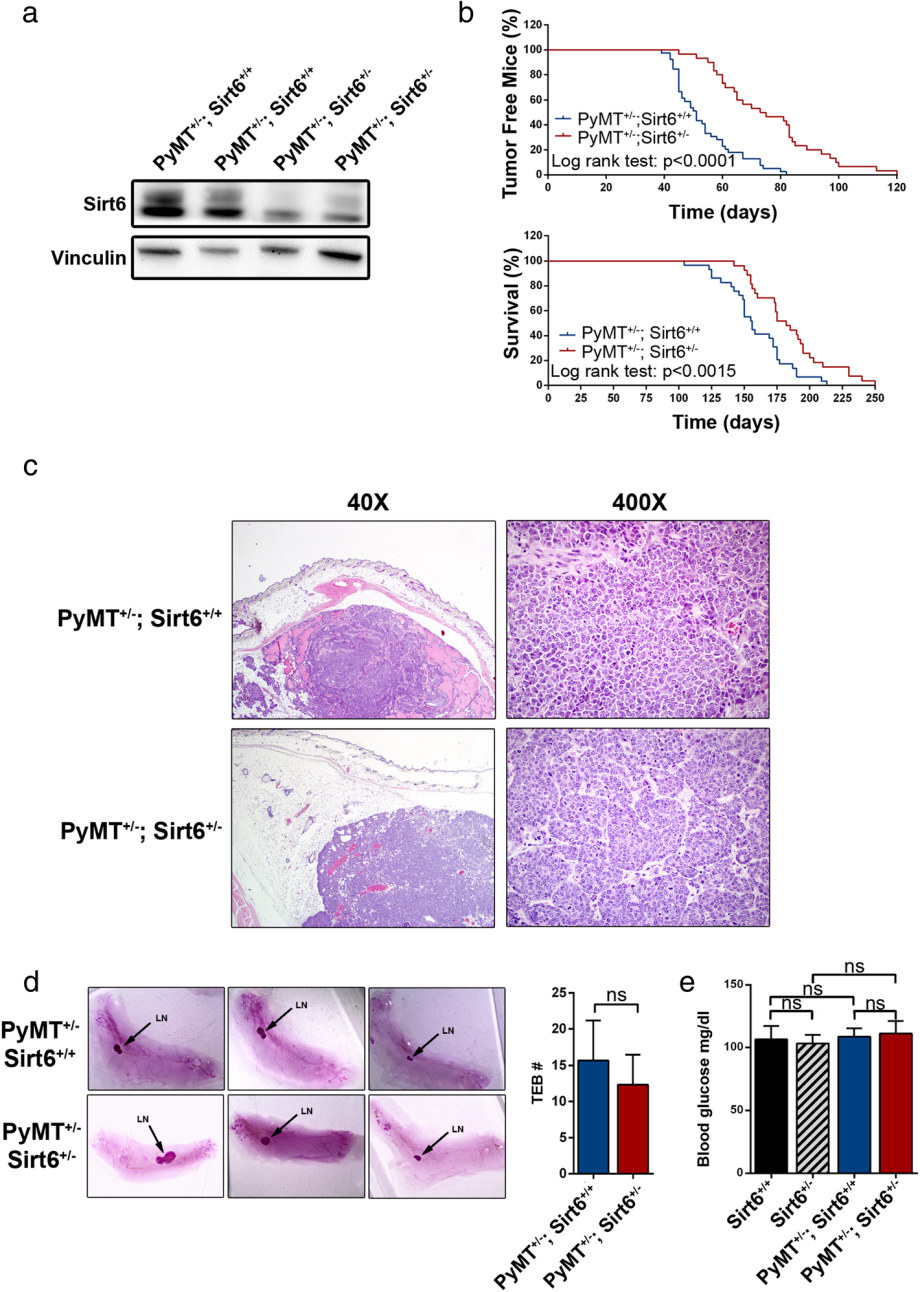
*Sirt6* heterozygous deletion increases tumor latency and enhances survival in the *MMTV-PyMT* mammary tumor model. **a** Tumor masses were excised from *MMTV-PyMT*^*+/−*^*; Sirt6*^*+/+*^ and *MMTV-PyMT*^*+/−*^*; Sirt6*^*+/−*^ mice; protein lysates were generated, and Sirt6 and vinculin levels were detected by Western blot. **b** Tumor latency (upper panel) and overall survival (lower panel) of *MMTV-PyMT*^*+/−*^*; Sirt6*^*+/+*^ (*n* = 39) and of *MMTV-PyMT*^*+/−*^*; Sirt6*^*+/−*^ (*n* = 30) mice were monitored over time. **c** Histopathological analysis of the mammary glands from *MMTV-PyMT*^*+/−*^*; Sirt6*^*+/+*^ and *MMTV-PyMT*^*+/−*^*; Sirt6*^*+/−*^ mice (hematoxylin-eosin; × 40 and × 400) shows poorly differentiated (G3) murine ductal carcinoma of the breast consisting in dysmetrical elements with solid growth, brisk mitotic activity, and scattered areas of necrosis in both types of tumor. **d** Mammary glands from 4-week-old *MMTV-PyMT*^*+/−*^*; Sirt6*^*+/+*^ (*n* = 3) and *MMTV-PyMT*^*+/−*^*; Sirt6*^*+/−*^ (*n* = 3) mice were stained by carmine alum (left) and used to quantify the TEB (right). LN, lymph node. Data are presented as mean ± SD; ns, not statistically significant. **e** Blood glucose levels in *Sirt6*^*+/+*^ (*n* = 6), *Sirt6*^*+/−*^ (*n* = 3), *MMTV-PyMT*^*+/−*^*; Sirt6*^*+/+*^ (*n* = 3), and *MMTV-PyMT*^*+/−*^*; Sirt6*^*+/−*^ (*n* = 6) mice. Data are presented as mean ± SD. ns, not statistically significant

In line with a previous study [31], *Sirt6*^*+/−*^ mice, which we typically maintained for several months, failed to show any adverse or debilitating phenotype. They remained fully viable and active up to more than 21 months of age (which is the longest observation period we recorded) and were phenotypically indistinguishable from the *Sirt6*^*+/+*^ animals. *MMTV-PyMT*^*+/−*^*; Sirt6*^*+/+*^ and *MMTV-PyMT*^*+/−*^*; Sirt6*^*+/−*^ animals were born at the expected Mendelian rate [49.7% *MMTV-PyMT*^*+/−*^*; Sirt6*^*+/+*^ mice (*n* = 149) and 50.7% *MMTV-PyMT*^*+/−*^*; Sirt6*^*+/−*^ (*n* = 152)]*.* The heterozygous *Sirt6* deletion resulted in a decreased Sirt6 protein expression in mammary tumor masses isolated from *MMTV-PyMT*^*+/−*^*; Sirt6*^*+/−*^ animals as compared to the control mice (Fig. 1a and Additional file 1: Fig. S1A). Mice carrying the heterozygous *Sirt6* deletion exhibited a marked increase in tumor latency and a consistent increase in their overall survival (Fig. 1b). No difference in terms of histology between mammary tumors from *MMTV-PyMT*^*+/−*^*; Sirt6*^*+/−*^ vs. *MMTV-PyMT*^*+/−*^*; Sirt6*^*+/+*^ mice was detected. In both cases, our pathology assessments demonstrated multifocal, highly fibrotic, adenocarcinomas involving the entire mammary fat pad (Fig. 1c). No difference between tumors from *MMTV-PyMT*^*+/−*^*; Sirt6*^*+/−*^ vs. *MMTV-PyMT*^*+/−*^*; Sirt6*^*+/+*^ mice was observed in terms of the number of mitoses [41.2 ± 16.6 and 33.5 ± 18.4 in *MMTV-PyMT*^*+/−*^*; Sirt6*^*+/+*^ (*n* = 6) and in *MMTV-PyMT*^*+/−*^*; Sirt6*^*+/−*^ (*n* = 4) mice, respectively; *P* = 0.53] and of microvessels [as detected by CD34 staining; 10.3 ± 8.5 and 12.8 ± 9.5 in *MMTV-PyMT*^*+/−*^*; Sirt6*^*+/+*^ (*n* = 6) and in *MMTV-PyMT*^*+/−*^*; Sirt6*^*+/−*^ (*n* = 4) mice, respectively; *P* = 0.7] per field. A non-significant trend towards an increase in the number of apoptotic tumor cells per field was observed as a result of *Sirt6* heterozygous deletion [5.7 ± 1 and 7.3 ± 1 in *MMTV-PyMT*^*+/−*^*; Sirt6*^*+/+*^ (*n* = 6) and in *MMTV-PyMT*^*+/−*^*; Sirt6*^*+/−*^ (*n* = 4) mice, respectively; *P* = 0.13].

Since previous studies indicated that the heterozygous deletion of another sirtuin family member, Sirt1, increases mammary tumor latency by interfering with the normal development of the mammary gland [32], we monitored the mammary gland development in *MMTV-PyMT*^*+/−*^*; Sirt6*^*+/+*^ and *MMTV-PyMT*^*+/−*^*; Sirt6*^*+/−*^ mice. In particular, we quantified the number of TEB, the key structures that regulate elongation and branching of the mammary gland into the fat pad and thereby drive ductal morphogenesis. Comparisons of 4-week-old *MMTV-PyMT*^*+/−*^*; Sirt6*^*+/+*^ and *MMTV-PyMT*^*+/−*^*; Sirt6*^*+/−*^ mice showed no significant difference in terms of number and size of TEB (Fig. 1d). Thus, the observed effect of *Sirt6* deletion on mammary tumorigenesis did not reflect impaired ductal morphogenesis in *MMTV-PyMT*^*+/−*^*; Sirt6*^*+/−*^ mice.

A homozygous deletion of *Sirt6* was previously reported to lower blood glucose levels due to increased expression of glucose transporters, such as GLUT4, and of increased glycolytic enzymes in many tissues [18]. Thus, because reduced circulating glucose levels could in principle account for the antitumor effect of SIRT6 depletion, we assessed whether the heterozygous *Sirt6* deletion would also lower blood sugar levels in mice. However, we could not detect any reduction in blood glucose levels in *MMTV-PyMT*^*+/−*^*; Sirt6*^*+/−*^ as compared to *MMTV-PyMT*^*+/−*^*; Sirt6*^*+/+*^ animals or in *Sirt6*^*+/−*^ compared to *Sirt6*^*+/+*^ mice (Fig. 1e). Consistent with this finding, we failed to detect increased *Glut4* and hexokinase 2 (*Hk2*) expression in several tissues, such as heart, lung, spleen, kidney, and mesenteric VAT of *MMTV-PyMT*^*+/−*^*; Sirt6*^*+/−*^ mice as compared to *MMTV-PyMT*^*+/−*^*; Sirt6*^*+/+*^ animals (Additional file 1: Fig. S1B). Therefore, reduced circulating glucose levels did not account for the anticancer effect observed in response to the heterozygous deletion of *Sirt6* in this BC model.

Overall, our results clearly indicate that reducing Sirt6 expression slows mammary cancer development in the mouse *MMTV-PyMT* BC model. These results are also consistent with those studies attributing an adverse prognostic significance to high SIRT6 levels in BC [16, 17].

### SIRT6 silencing reduces tumor growth in MDA-MB-231 xenografts

**Fig. 2:**
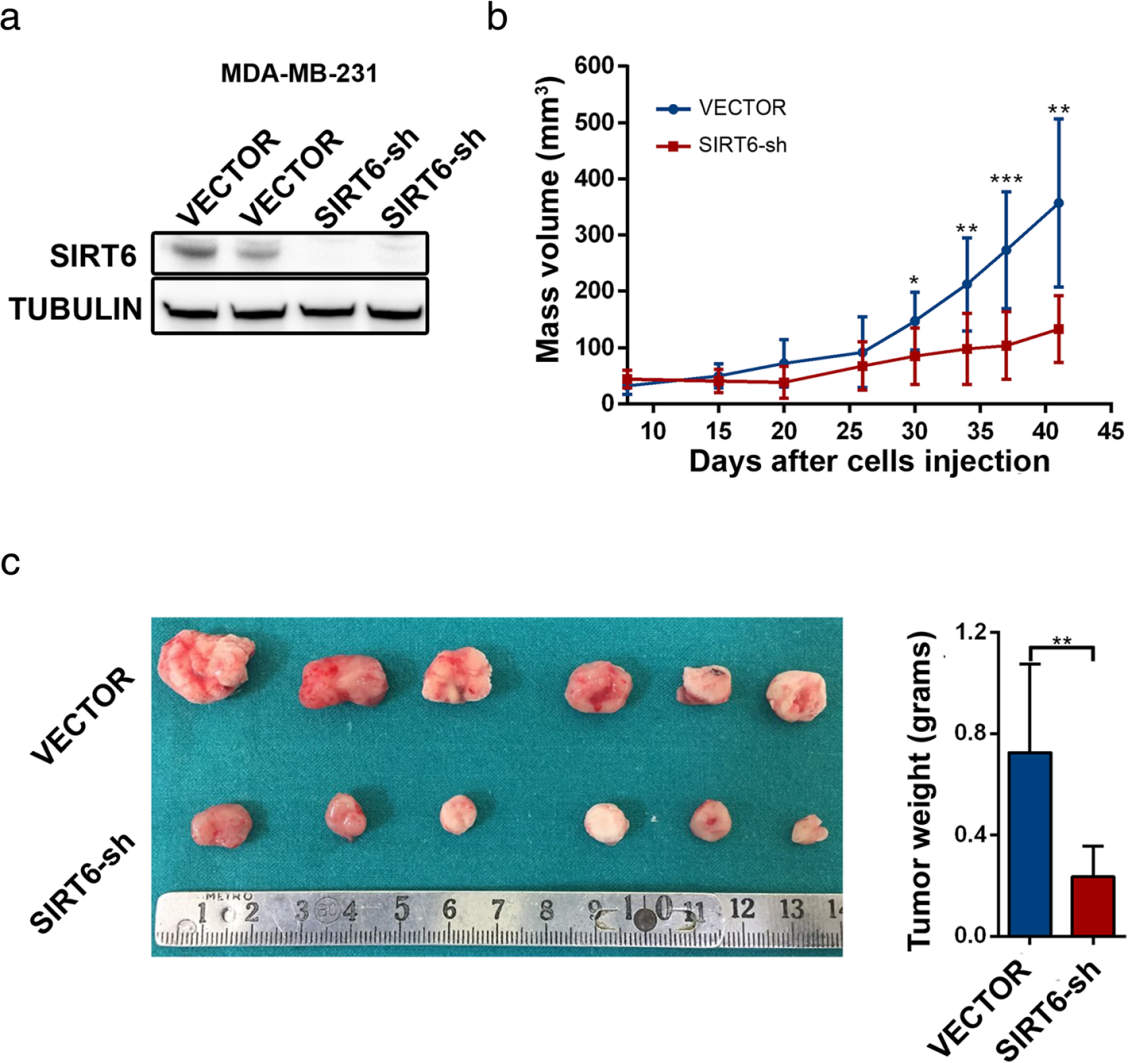
SIRT6 silencing reduces tumor growth in MDA-MB-231 xenografts. **a**, **b** 2 × 10^6^ MDA-MB-231 BC cells transduced with either a SIRT6-shRNA or a control vector were injected subcutaneously in both flanks of BALB/c athymic nude mice (Hsd:Athymic Nude-*Foxn1*^*nu*^) (VECTOR, *n* = 8 and SIRT6-sh, *n* = 10). In **a**, mice were sacrificed when the tumors became palpable; tumors were used for protein lysate generation, and SIRT6 and α-tubulin were detected by Western blot. In **b**, tumor volume was monitored over time from the day of tumor cell inoculation. Data are presented as mean ± SD. *P < 0.05, **P < 0.01, ***P < 0.001 (statistical values refer to the comparison between tumors with silenced SIRT6 and control tumors at the same time point). **c** Fifty days after tumor  cell inoculation, mice were sacrificed, and masses were collected, imaged, and weighted (VECTOR: *n* = 6; SIRT6-sh: *n* = 6). Data are presented as mean ± SD. **P < 0.01

Since in *MMTV-PyMT*^*+/−*^*; Sirt6*^*+/−*^ mice, *Sirt6* deletion affects all bodily tissues, the observed delay in tumor development and the corresponding enhancement of mouse survival could in principle reflect non-cell-autonomous anticancer effects (i.e., effects unrelated to *Sirt6* deletion in tumor cells). To address this possibility, we silenced SIRT6 by RNA interference in the BC cell line MDA-MB-231 (Fig. 2a), injected these cells (or cells harboring a control shRNA) subcutaneously into both flanks of nude mice, and monitored tumor growth. As shown in Fig. 2b, tumor masses developed from SIRT6-silenced MDA-MB-231 cells exhibited a markedly reduced growth, which was also confirmed by isolating the tumor masses at the end of the experiment and by measuring their size and weight (Fig. 2c). These results are consistent with reduced SIRT6 levels slowing BC growth in a cell-autonomous fashion, although in principle, the possibility that *Sirt6* depletion also causes non-cell-autonomous anti-tumor effects in the *MMTV-PyMT* mammary tumor model cannot be excluded.

### SIRT6 enhances OXPHOS in mouse mammary tumors and in human BC cells

Studies showed that tumor growth requires active mitochondrial aerobic metabolism to support cancer cell proliferation, cancer stem cell survival, and metastasis [3, 33]. Consistent with this notion, agents targeting mitochondrial respiratory complexes, such as metformin (which inhibits complex I activity) show promising anticancer properties [26, 34].

**Fig. 3:**
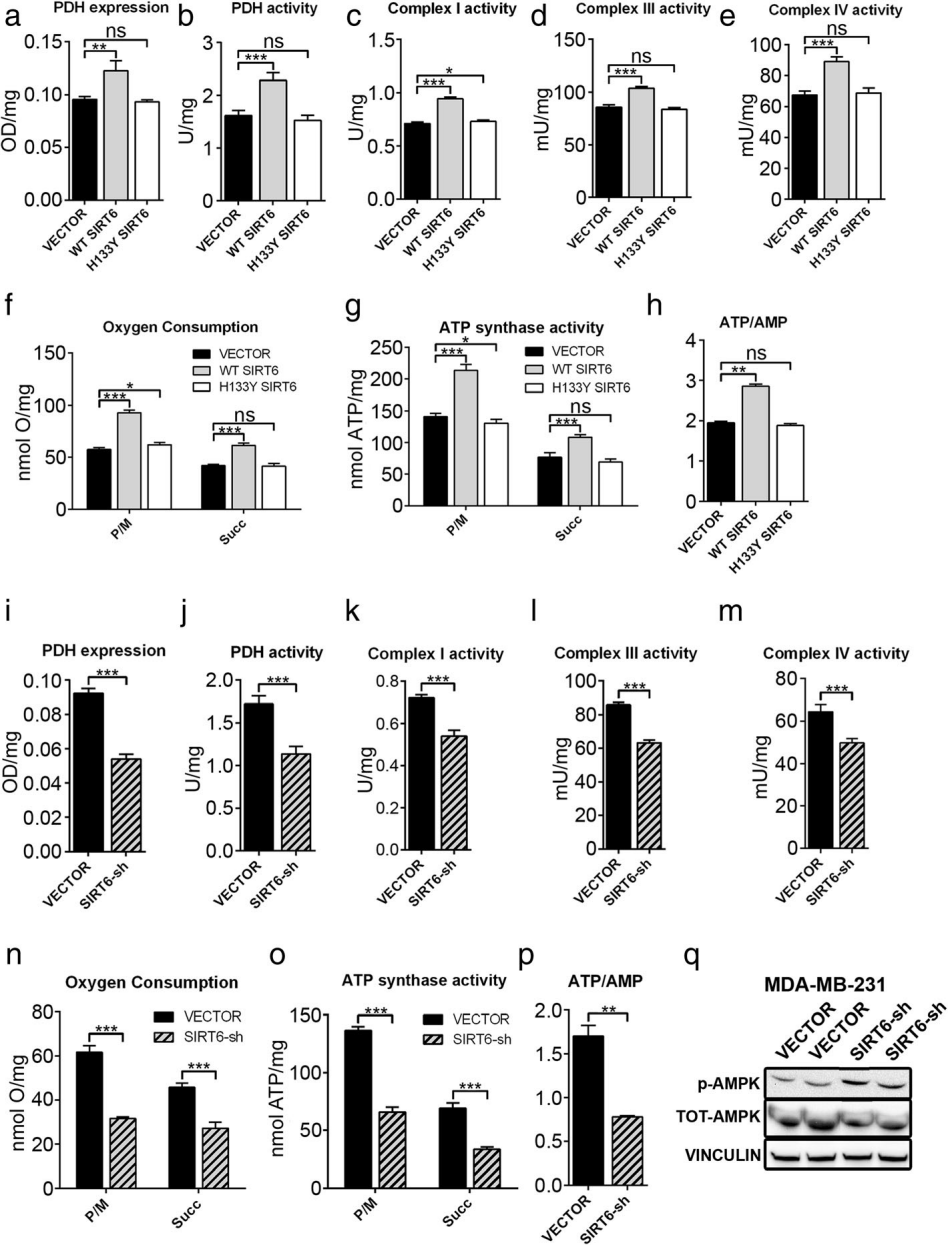
SIRT6 enhances OXPHOS and energy status in MDA-MB-231 cells. **a**–**h** Expression (**a**) and activity (**b**) of pyruvate dehydrogenase (PDH), activity of mitochondrial complexes (**c**–**e**), oxygen consumption (**f**), activity of F_o_-F_1_ ATP synthase (**g**), and energy status, expressed as ATP/AMP ratio (**h**), were measured in MDA-MB-231 cells transduced with human WT or catalytically inactive (H133Y) SIRT6, or with a control vector (VECTOR). Data are presented as mean ± SD of three different experiments. *P < 0.05, **P < 0.01, ***P < 0.001; ns, not statistically significant. **i**–**p** Expression (**i**) and activity (**j**) of PDH, activity of mitochondrial complexes (**k**–**m**), oxygen consumption (**n**), activity of F_o_-F_1_ ATP synthase (**o**), and energy status, expressed as ATP/AMP ratio (**p**) were measured in MDA-MB-231 cells transduced with a short hairpin RNA targeting SIRT6 or with a control vector (VECTOR). Data are presented as mean ± SD of three different experiments. **P < 0.01, ***P < 0.001. **q** 2 × 10^6^ MDA-MB-231 BC cells transduced with either a SIRT6-shRNA or a control vector (VECTOR) were injected subcutaneously in both flanks of BALB/c athymic nude mice. Mice were sacrificed, and tumors were excised 50 days after cell inoculation. Proteins were extracted, and phosphorylated AMPK (Thr183, Thr172), total AMPK, and vinculin were detected by Western blot. One representative experiment out of two is presented

**Fig. 4:**
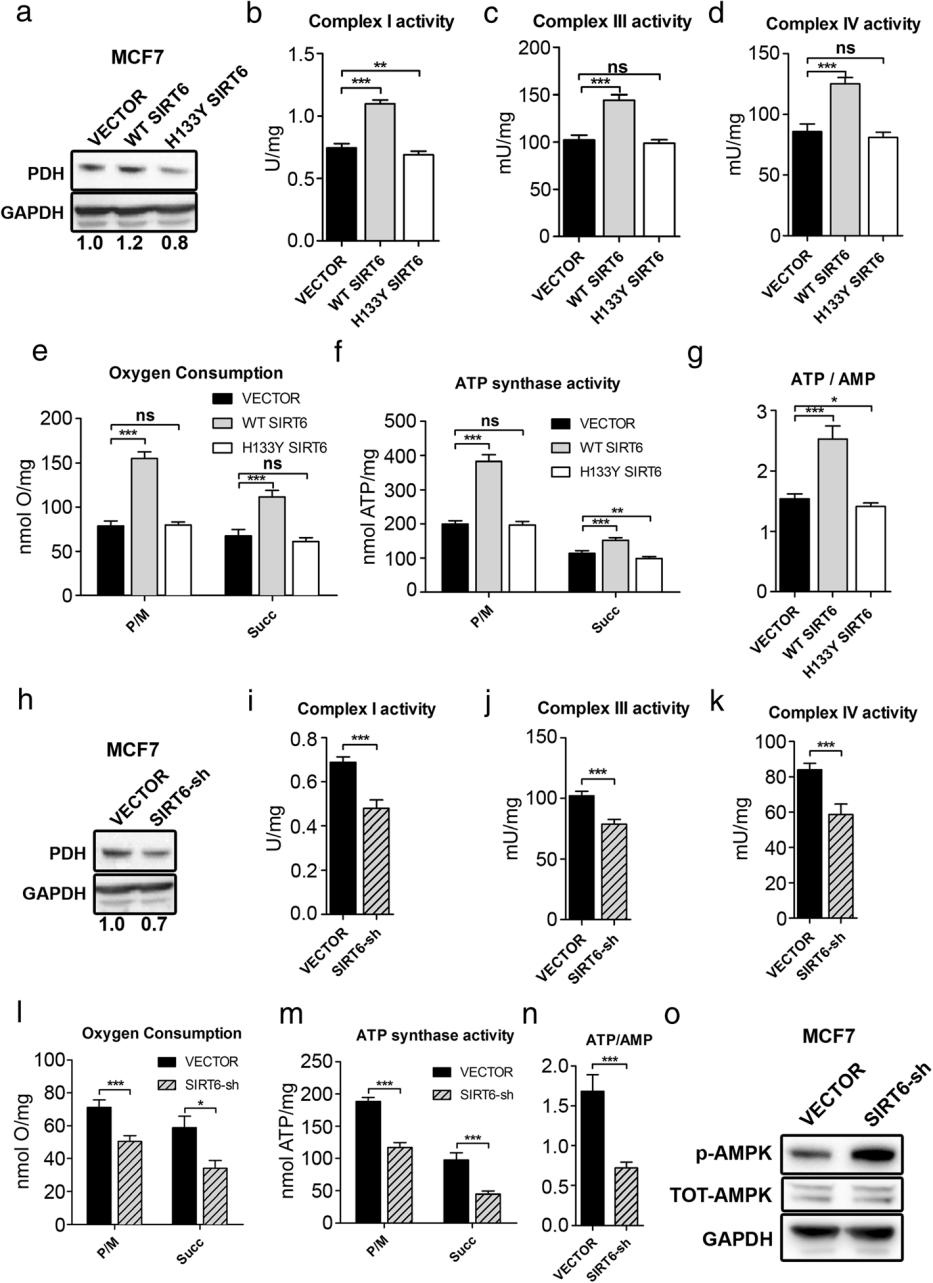
SIRT6 enhances OXPHOS and energy status in MCF7 cells. **a** Western blot analysis of PDH expression in MCF7 cells transduced with human WT or catalytically inactive (H133Y) SIRT6 or with a control vector (VECTOR). PDH expression was quantified following normalization to GAPDH. **b-g** Activity of mitochondrial complexes (**b**–**d)**, oxygen consumption (**e**), activity of F_o_-F_1_ ATP synthase (**f**), and energy status, expressed as ATP/AMP ratio (**g**), were measured in MCF7 cells transduced with human WT or catalytically inactive (H133Y) SIRT6 or with a control vector (VECTOR). Data are presented as mean ± SD of three different experiments. *P < 0.05, **P < 0.01, ***P < 0.001; ns, not statistically significant. **h** Western blot analysis of PDH expression in MCF7 cells transduced with an shRNA targeting SIRT6 (SIRT6-sh) or with a control vector (VECTOR). PDH expression was quantified following normalization to GAPDH. **i**–**n** Activity of mitochondrial complexes (**i**–**k**), oxygen consumption (**l**), activity of F_o_-F_1_ ATP synthase (**m**), and energy status, expressed as ATP/AMP ratio (**n**), were measured in MCF7 cells transduced with an shRNA targeting SIRT6 (SIRT6-sh) or with a control vector (VECTOR). Data are presented as mean ± SD of three different experiments. *P < 0.05, ***P < 0.001. **o** Protein lysates were generated from MCF7 that were engineered with a control vector (VECTOR) or with an shRNA targeting SIRT6 (SIRT6-sh)\. Phosphorylated (Thr183, Thr172) and total AMPK as well as GAPDH were detected by Western blot (one representative experiment out of three is presented)

**Fig. 5:**
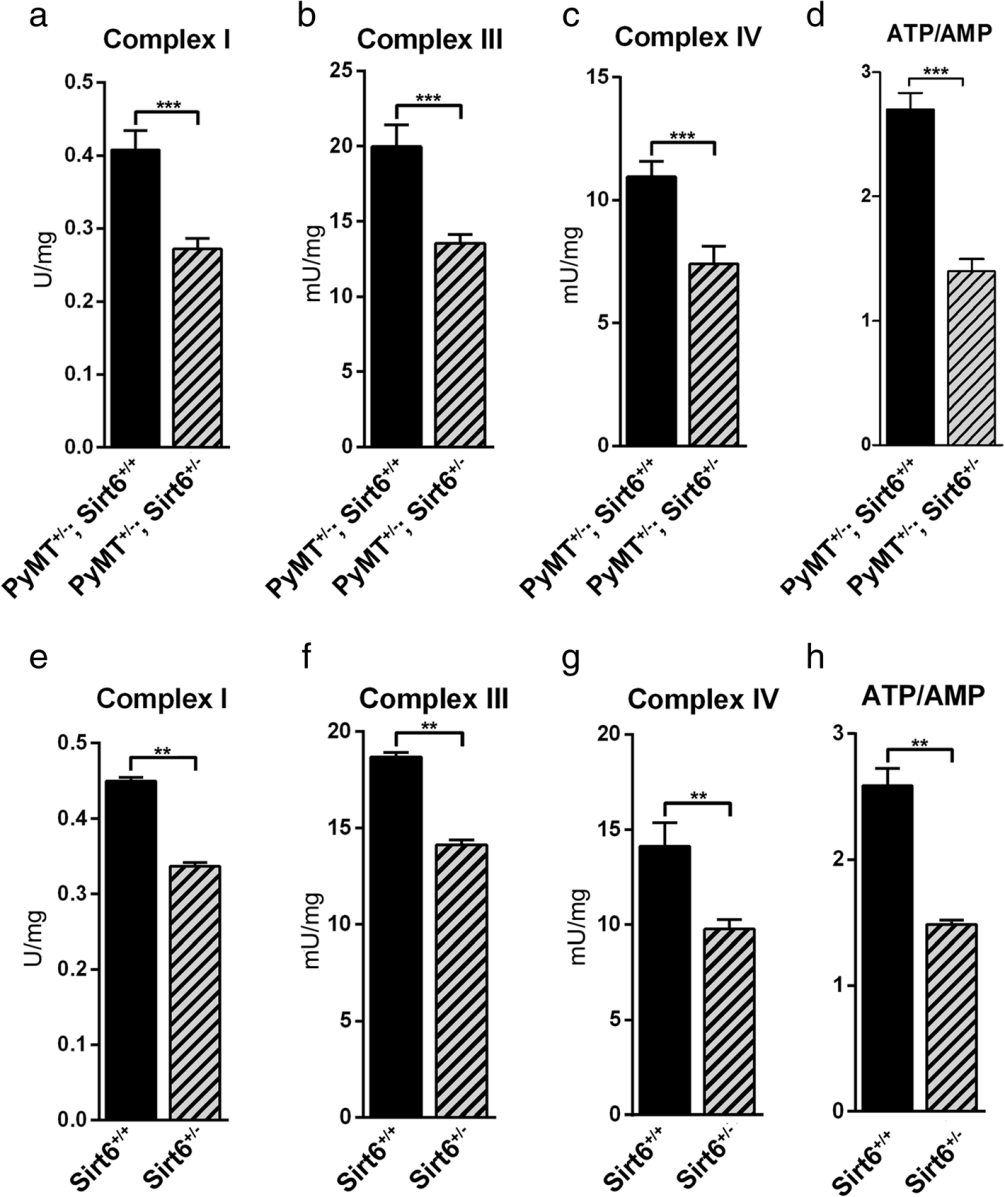
*Sirt6* heterozygous deletion decreases OXPHOS and ATP/AMP ratio in MMTV-PyMT mammary tumors and in healthy mammary glands. **a–d** Mitochondrial complex activity (**a–c**) and energy status, expressed as ATP/AMP ratio (**d**), were measured in tumor masses that were isolated from 12-week-old *MMTV-PyMT*^*+/−*^*; Sirt6*^*+/+*^ (*n* = 5) and *MMTV-PyMT*^*+/−*^*; Sirt6*^*+/−*^ (*n* = 4) mice. Data are presented as mean ± SD. ***P < 0.001. **e**–**h** Mitochondrial complex activity (**e**–**g)** and energy status, expressed as ATP/AMP ratio (**h**), were measured in the mammary glands that were isolated from 12-week-old *Sirt6*^*+/+*^ (*n* = 4) and *Sirt6*^*+/−*^ (*n* = 4) mice. Data are presented as mean ± SD. **P < 0.01

Previous studies suggested that OXPHOS may be reduced as a consequence of decreased SIRT6 levels [18, 35]. Thus, we hypothesized that blunting SIRT6 expression could affect OXPHOS and cellular energy status in BC cells. To address this hypothesis, we used MDA-MB-231 and MCF7 cells, after transducing them with either WT or catalytically inactive (H133Y) SIRT6 (Additional file 2: Fig. S2A, B). In these engineered cells, we measured the expression and activity of PDH, the activity of respiratory complexes I, III, and IV; O_2_ consumption; ATP synthase activity; and ATP/AMP ratio. Overexpression of WT, but not of catalytically inactive, SIRT6 enhanced the expression and activity of PDH, the activity of respiratory complexes, O_2_ consumption, and ATP synthesis, determining a consistent increase of the ATP/AMP ratio (Figs. 3a–h and 4a–g) in both cell lines. Opposite effects were obtained by SIRT6 silencing in MDA-MB-231 and in MCF7 cells (Figs. 3i–p and 4 h–n and Additional file 2: Fig. S2C, D). Consistent with these data obtained in human BC cell lines, mammary tumors from *MMTV-PyMT*^*+/−*^*; Sirt6*^*+/−*^ mice exhibited decreased complex I, III, and IV activity as well as reduced ATP/AMP ratio as compared to tumors from *MMTV-PyMT*^*+/−*^*; Sirt6*^*+/+*^ mice (Fig. 5a–d). We also isolated the mammary glands from regular *Sirt6*^*+/+*^ or *Sirt6*^*+/−*^ 129/Ola mice which had not been crossed with *MMTV-PyMT* animals and subjected them to the same biochemical analyses. Decreased respiratory complex activity and ATP/AMP ratio were also detected in healthy, non-tumor-bearing mammary glands from *Sirt6*^*+/−*^ mice as compared to the glands from *Sirt6*^*+/+*^ animals (Fig. 5e–h). Therefore, these data indicate that the reduction in OXPHOS through SIRT6 depletion is not restricted to transformed mammary tissue. Rather, it appears to be also present in healthy mammary glands, likely contributing to the delay in the development of *PyMT*-induced tumors.

Homozygous Sirt6 deletion was previously reported to reduce the expression of the OXPHOS-related genes ubiquinol-cytochrome c reductase, Rieske iron-sulfur polypeptide 1 (Uqcrfs1), cytochrome c oxidase subunit 5B (Cox5b), NADH:ubiquinone oxidoreductase subunit B8 (Ndufb8), ubiquinol-cytochrome c reductase core protein 2 (Uqcrc2), and succinate dehydrogenase complex iron sulfur subunit B (Sdh) in mouse skeletal muscle tissue [36]. Thus, we evaluated whether similar changes would also occur in BC cells in response to reduced SIRT6, possibly contributing to the observed effects of SIRT6 modulation on OXPHOS. *UQCRFS1*, *COX5B*, and *NDUFB8* expression was blunted by both SIRT6 silencing in MDA-MB-231 cells and by the *Sirt6* heterozygous deletion in mouse mammary tumors (Additional file 3: Fig. S3A, B). *UQCRC2* expression was reduced by SIRT6 silencing in MDA-MB-231, but not in *Sirt6*^*+/−*^ mouse tumor masses. Finally, *SDH* was not affected by either SIRT6 silencing in MDA-MB-231 cells or by *Sirt6* deletion in mouse mammary neoplasms. Therefore, these data are consistent with SIRT6 also promoting the expression of genes encoding for respiratory chain proteins, such as *UQCRFS1*, *COX5B*, *NDUFB8*, and *UQCRC2*, in BC cells.

We also evaluated whether the changes in OXPHOS we observed in response to different SIRT6 levels in BC cells would reflect differences in mitochondrial mass or in mitochondrial morphology. To this end, we first stained MDA-MB-231 with the mitochondrial dye Mitotracker deep red and analyzed them by flow cytometry (Additional file 3: Fig. S3C). However, according to this approach, SIRT6 overexpression and SIRT6 silencing failed to increase and to decrease, respectively, the mitochondrial mass. Similar results were obtained by monitoring the ratio between mitochondrial and nuclear DNA in MDA-MB-231 cells: neither SIRT6 overexpression nor its silencing affected such ratio (Additional file 3: Fig. S3D). In subsequent experiments, MDA-MB-231 cells overexpressing SIRT6 or silenced for this protein were stained with Mitotracker deep red and analyzed by confocal microscopy. Here, again, we failed to detect significant changes in any of the morphological parameters that we analyzed, including mitochondrial area, perimeter, circularity, and solidity in response to different SIRT6 levels (Additional file 3: Fig. S3E and data not shown). Therefore, overall, these data indicate that the effects of SIRT6 overexpression and of its silencing on OXPHOS in BC cells are not justified by corresponding changes in mitochondrial mass or morphological features.

Finally, in the attempt to define the mechanism underlying the decreased PDH activity in tumors from *MMTV-PyMT*^*+/−*^*; Sirt6*^*+/−*^ mice, we investigated whether this would reflect an increased expression of piruvate dehydrogenase kinase 1 (*PDK1*) or piruvate dehydrogenase kinase 4 (*PDK4*) (which both catalyze inhibitory phosphorylation of PDH), as previously reported as a consequence of reduced Sirt6 in intestinal cancer and in the heart, respectively [11, 37]. However, neither of these two genes was increased in its expression by the heterozygous *Sirt6* deletion (Additional file 4: Fig. S4A). Similarly, no increase in *PDK1* and *PDK4* expression was detected in MDA-MB-231 cells as a result of SIRT6 silencing (data not shown). On the other hand, we found that, consistent with our previous data in pancreatic cancer cells [14], SIRT6 overexpression increased, while SIRT6 silencing decreased, intracellular Ca^2+^ concentration in MDA-MB-231 cells (Additional file 4: Fig. S4B). Thus, since intracellular Ca^2+^ enhances PDH activity [38, 39], SIRT6’s ability to regulate these cation levels could account (together with the increased PDH expression) for the enhanced PDH function we observed in SIRT6-overexpressing BC cells.

Overall, these findings indicate that SIRT6 depletion reduces OXPHOS and energy status in BC cells of a murine and human source and point to a probable mechanism for the reduced tumor growth occurring in response to SIRT6 depletion.

### SIRT6 regulates AMPK activity in BC cells

The ATP/AMP ratio is the main determinant of AMP-activated protein kinase (AMPK) activation [40]. In turn, activated AMPK is responsible for orchestrating a tumor-suppressive response that includes a mammalian target of rapamycin (mTOR) inhibition and autophagy initiation [41]. We found that MCF7 and MDA-MB-231 cells with silenced SIRT6 exhibited increased levels of AMPK phosphorylation (Fig. 4o and Additional file 5: Fig. S5A). Accordingly, higher AMPK activation was also observed in xenografts of MDA-MB-231 with silenced SIRT6 as compared to xenografts of control cells (Fig. 3q and Additional file 5: Fig. S5B). Therefore, these findings are in line with SIRT6 depletion causing energetic stress in BC cells and consequently increasing AMPK activity.

## Discussion

Here, we show that a heterozygous deletion of the *Sirt6* gene increases tumor latency and improves overall survival in the MMTV-PyMT mouse model. Such an effect is recapitulated by SIRT6 silencing in MDA-MB-231 xenografts, indicating a cell-autonomous anticancer effect of SIRT6 depletion in BC cells. We show that SIRT6 boosts OXPHOS and enhances energy status in BC cells and that this translates into dampened AMPK activity (Additional file 6: Fig. S6). Our in vivo data, showing delayed mammary tumor development and increased survival in *MMTV-PyMT*^*+/−*^*; Sirt6*^*+/−*^ mice as compared to *MMTV-PyMT*^*+/−*^*; Sirt6*^*+/+*^ animals, are in line with the previously reported adverse prognostic significance of high SIRT6 expression in BC [16, 17]. These two studies attributed the “pro-oncogenic” role of SIRT6 in BC not only to the ability of SIRT6 to promote DNA repair in BC cells and, consequently, to mediate resistance to chemotherapeutics, but also to the increased expression of MMP9, β-catenin, CCND1, and NF-κB and to enhanced BC cell migration and invasiveness. Our data point out a new “metabolic” function of this NAD^+^-dependent deacetylase in BC cells, which consists of SIRT6’s ability to enhance PDH expression and activity, OXPHOS, and ATP availability in BC cells. Quite remarkably, SIRT6 overexpression also increased oxygen consumption and ATP synthase activity in MDA-MB-231 cells, which is normally considered to be a glycolysis-dependent cell line, as opposed to other BC cells, which primarily rely on OXPHOS for their metabolism, including MCF7 cells [42].

Studies show that, despite early models proposing that cancer cells would primarily rely on aerobic glycolysis for their survival, mitochondrial function is actually decisive in many neoplasms. Loss of the tumor suppressor RB1, but also several proto-oncogenes, such as mitochondrial STAT3, FER, and its variant, FerT, and CHCHD2, induces OXPHOS [3]. Accordingly, OXPHOS inhibitors, such as metformin, tigecycline, or salinomycin, hold promise for preventing or treating different forms of cancer, including BC. Our study indicates SIRT6 as a new, potentially druggable target [43–46] to be exploited for interfering with OXPHOS in BC, thereby achieving cancer-preventive but also, possibly, therapeutic effects.

Previous studies attributed SIRT6’s ability to enhance OXPHOS to SIRT6-mediated reduction of PDK1 and PDK4 expression, which in turn would increase PDH activity [11, 37]. As opposed to these studies, in mammary tumors from *MMTV-PyMT*^*+/−*^*; Sirt6*^*+/−*^ mice and in MDA-MB-231 cells with silenced SIRT6, we did not detect increased *PDK1* or *PDK4* expression. However, we did find SIRT6 to regulate PDH expression. Specifically, SIRT6 overexpression increased, while its silencing decreased, PDH levels. In addition, consistent with a previous study by our group in pancreatic cancer cells [14], we found SIRT6 to increase the intracellular concentration of Ca^2+^, which is a known enhancer of PDH function [38, 39], in MDA-MB-231 cells. Thus, we propose that SIRT6 may promote PDH activity in BC cells via at least two mechanisms: (i) by increasing PDH levels and (ii) by enhancing the intracellular Ca^2+^ concentration. Conceivably, by blunting PDH expression and activity, SIRT6 depletion [and possibly SIRT6 inhibition via small molecules [45]] could lead to lower levels of acetyl-CoA and TCA cycle intermediates in BC cells. Thus, it is possible that cancer cells with low SIRT6 will show increased uptake of other mitochondrial fuels, such as glutamine and fatty acids. Future studies should address this possibility as a way to identify metabolic liabilities of cancer cells with low SIRT6 expression or in which SIRT6 is pharmacologically inhibited.

A previous study also reported that the expression of the OXPHOS-related genes *Uqcrfs1*, *Cox5b*, *Ndufb8*, *Uqcrc2*, and *Sdh* was markedly reduced in *Sirt6*^*−/−*^ skeletal muscle tissue [36]. In line with this study, we found *UQCRFS1*, *COX5B*, and *NDUFB8* expression to be reduced by both SIRT6 silencing (in MDA-MB-231 cells) and *Sirt6* heterozygous deletion (in tumors from *MMTV-PyMT*^*+/−*^*; Sirt6*^*+/−*^ mice). *UQCRC2* expression was also lower in MDA-MB-231 cells with silenced SIRT6 as compared with the control cells. *SDH* was neither reduced by SIRT6 silencing in MDA-MB-231 cells nor in tumors from *MMTV-PyMT*^*+/−*^*; Sirt6*^*+/−*^ mice. Thus, these data indicate that SIRT6-mediated regulation of OXPHOS in BC cells probably also relies on SIRT6 ability to regulate the amounts of respiratory chain proteins in addition to its effect on PDH expression and activity. Finally, we previously reported that SIRT6-overexpressing MCF7 cells have higher levels of NAD(H) and of NADPH, which are both important coenzymes participating in various energy metabolism pathways [44]. These findings are also in line with the results of this study and suggest that the enhanced energy status of SIRT6-overexpressing BC cells could also reflect higher NAD(P)(H) availability.

The heterozygous deletion of *Sirt6* also reduced complex I, III, and IV activity and ATP/AMP ratio in healthy (non-PyMT-transformed) mammary glands, indicating that the effect of reduced SIRT6 on mammary gland cell metabolism is independent of whether the tissue is transformed or not. Based on these data, one could argue that reduced ATP availability in the mammary gland may hinder tumor development starting from its very early stages, thus justifying the marked extension in tumor latency observed in *MMTV-PyMT*^*+/−*^*; Sirt6*^*+/−*^ mice.

In our hands, SIRT6 depletion resulted in increased AMPK activation as detected by its phosphorylation on threonines 172 and 183. These findings, which we attribute to the low ATP/AMP ratio that we observed in response to reduced SIRT6, are consistent with those of Ming and colleagues, who also found such an increase in AMPK phosphorylation upon SIRT6 silencing in skin cancer cells [47]. On the other hand, Elhanati and colleagues found that, in the liver, SIRT6 overexpression, rather than its downregulation, decreases ATP/AMP ratio and activates AMPK [48]. This raises the interesting possibility that SIRT6 effects on AMPK activity may vary between different tissues and reflect different regulatory mechanisms.

Curiously, a previous study evaluated the effect of SIRT6 overexpression (using Sirt6BAC mice) in the MMTV-PyMT mammary tumor model and found that SIRT6 overexpression also has the ability to hamper tumor progression in mice [49]. According to this study, the antitumor activity of SIRT6 overexpression would be restricted to BC with constitutively active PI3K and result from SIRT6 ability to reduce PI3K signaling as well as BC stem cell-like characteristics. In addition, the anticancer activity of overexpressed SIRT6 was shown to be independent of SIRT6 deacetylase activity. Our data complement and extend these findings and indicate that both SIRT6 depletion and SIRT6 overexpression probably result in suboptimal conditions for BC development. SIRT6 depletion has anticancer effects in BC very likely through its ability to reduce the expression and activity of PDH and OXPHOS and to cause energy stress in cancerous and pre-cancerous lesions. These effects are dependent on SIRT6 deacetylase activity, but independent of PI3KCA mutational status, since SIRT6 depletion had the same effect on OXPHOS-related parameters in MDA-MB-231 cells, which have WT PI3K, and in MCF7, which have a PIK3CA activating mutation [50]. On the other hand, in breast tumors with mutated PI3KCA, SIRT6 overexpression inhibits PI3K signaling in a deacetylase activity-independent fashion and thereby affects the cancer stem cell compartment.

## Conclusion

Although other mechanisms, such as impaired DNA repair ability and reduced β-catenin, cyclin D1 or NF-κB activity could also have contributed to the antitumor effects of low SIRT6 in our BC models, as suggested by earlier reports [16, 17], our findings complement these previous studies and depict a novel, key role for SIRT6 in BC metabolism. These new insights strengthen the rationale for targeting this deacetylase as a way to prevent or treat BC.

## Supplementary Information

**Additional file 1: Fig. S1.** Effect of *Sirt6* heterozygous deletion on *Sirt6*, *Glut4* and *Hk2* expression in mouse mammary tumors and tissues. A, B, Mammary tumors and healthy tissues [heart, lung, spleen, kidney and visceral adipose tissue (VAT)] were isolated from twelve-week-old and from four-week-old *MMTV-PyMT*^*+/-*^; *Sirt6*^*+/+*^ (*n* = 3) and *MMTV-PyMT*^*+/-*^; *Sirt6*^*+/-*^ (*n* = 3) mice, respectively, and used for protein lysate generation and for RNA extraction. In A, Sirt6 and vinculin levels were assessed by Western blot in mammary tumors. In B, *Hk2* and *Glut4* expression in bodily tissues was detected by QPCR. Data are presented as mean ± SD. ns: not statistically significant.

**Additional file 2: Fig. S2.** SIRT6 silencing and overexpression in MDA-MB-231 and MCF7 cell lines. A, B, Western blot analysis of MDA-MB-231 (A) and MCF7 (B) cell lines overexpressing wild type (WT) or catalytically inactive (H133Y) SIRT6 with respect to control cells (VECTOR). C, D, Western blot analysis of MDA-MB-231 (C) and MCF7 (D) cell lines silenced for SIRT6 (SIRT6-sh) with respect to control cells (VECTOR). A-D, One representative experiment out of three is presented.

**Additional file 3: Fig. S3.** Effect of SIRT6 deletion and overexpression on the expression of genes encoding for  respiratory chain proteins and on mitochondrial mass and features. A, B, RNA was extracted from MDA-MB-231 cells engineered with a control vector (VECTOR) or a SIRT6-targeting shRNA (SIRT6-sh) (A) and from *MMTV-PyMT*^*+/-*^; *Sirt6*^*+/+*^ (*n* = 6) and *MMTV-PyMT*^*+/-*^; *Sirt6*^*+/-*^ (*n* = 5) mice (B) and *UQCRFS1, COX5B, NDUFB8, UQCRC2* and *SDH* expression was determined by QPCR. In A, data are presented as mean ± SD of three different experiments. *p<0.05, **p<0.01, ns: not statistically significant. C, D, MDA-MB-231 cells were engineered to overexpress WT SIRT6 (or a control vector) or to express a SIRT6-shRNA (or a control vector). Thereafter, cells were stained with Mitotracker deep red and analyzed by flow cytometry to detect their mitochondrial mass (C) or used for DNA extraction (D). In D*,* the amounts of DNA coding for *tRNALeu* and *B2M* were used to quantify mitochondrial and nuclear DNA, respectively, and their ratio was calculated. In C, one representative experiment out of three is presented. In D, data are presented as mean ± SD of three different experiments. ns: not statistically significant. (E) MDA-MB-231 cells that were engineered to overexpress WT SIRT6 (or a control vector) or a SIRT6-shRNA (or a control vector) were stained with Mitotracker deep red and analyzed by confocal microscopy, estimating mitochondrial area and circularity. Panel on the left shows one representative experiment out of three. In the histograms, data are presented as mean ± SD of at least three biological replicates. ns: not statistically significant.

**Additional file 4: Fig. S4.** SIRT6 levels do not affect Pdk1/4 expression but regulate intracellular calcium concentration in breast cancer cells. A, RNA was extracted from mammary tumors from twelve-week-old *MMTV-PyMT*^*+/-*^; *Sirt6*^*+/+*^ (*n* = 6) and *MMTV-PyMT*^*+/-*^; *Sirt6*^*+/-*^ (*n* = 5) mice and *Pdk1* and *Pdk4* expression was determined by QPCR. B, MDA-MB-231 were engineered to overexpress WT SIRT6 (or a control vector) or to express a SIRT6-targeting shRNA (or a control vector). Thereafter, cells were used to measure intracellular calcium concentration. Data are presented as mean ± SD of three separate experiments. *p<0.05, ns: not statistically significant.

**Additional file 5: Fig. S5.** SIRT6 silencing enhances AMPK phosphorylation in MDA-MB-231 xenografts. A, MDA-MB-231 cells were engineered to express a SIRT6-shRNA (or a control vector). Thereafter, cells were used for protein lysate generation and phosphorylated AMPK (Thr183, Thr172), total AMPK and GAPDH were detected by Western blot. One representative experiment out of three is presented. B, MDA-MB-231 BC cells transduced with either a SIRT6-shRNA or with a control vector were injected subcutaneously into both flanks of BALB/c athymic nude mice. Animals were sacrificed 50 days after cell inoculation; tumors were used for protein lysate generation and phosphorylated and total AMPK, SIRT6 and vinculin were detected by Western blot. In the right panel, the intensity of the phospho-AMPK bands was normalized to that of the total AMPK bands and the phospho-AMPK/total-AMPK ratio in tumors with silenced SIRT6 was compared to that detected in control tumors. Data are presented as mean ± SD. *p<0.05.

**Additional file 6: Fig. S6.** Putative model for the metabolic, pro-oncogenic role of SIRT6 in breast tumorigenesis. SIRT6 increases expression and activity of PDH, as well as the levels of mitochondrial respiratory chain proteins. This results in increased OXPHOS and in a higher ATP/AMP ratio in BC cells. Ultimately, as a result of SIRT6 activity, AMPK activation is prevented and more ATP is available for cancer cells to grow.

**Additional file 7: Supplementary Table 1.** QPCR primer list.

## Data Availability

All data generated or analyzed during this study are included in this published article and its supplementary information files.

## Abbreviations

SIRT6: Sirtuin 6
BC: Breast cancer
PyMT: Polyoma middle tumor-antigen
MMTV-PyMT: Mouse mammary tumor virus-polyoma middle tumor-antigen
ATP: Adenosine triphosphate
AMP: Adenosine monophosphate
AMPK: AMP-activated protein kinase
OXPHOS: Oxidative phosphorilation
CCND1: Cyclin D1
MMP9: Matrix metalloproteinase 9
WT: Wild-type
DNA: Deoxyribonucleic acid
SDS-PAGE: Sodium dodecyl sulpahte-polyacrilamide gel electrophoresis
PVDF: Polyvinylidene fluoride
GAPDH: Glyceraldehyde 3-phosphate dehydrogenase
EDTA: Ethylenediaminetetraacetic acid
RT: Room temperature
QPCR: Quantitative real-time polymerase chain reaction
cDNA: Complementary DNA
VAT: Visceral adipose tissue
FACS: Fluorescence-activated cell sorting
MFI: Mean fluorescence intensity
mtDNA: Mitochondrial DNA
nDNA: Nuclear DNA
KO: Knock out
PBS: Phosphate-buffered saline
NADP^+^: Nicotinamide adenine dinucleotide phosphate
HK: Hexokinase
G6PD: Glucose-6-phosphate dehydrogenase
NADH: Nicotinamide adenine dinucleotide reduced
AK: Adenylate kinase
PK: Pyruvate kinase
LDH: Lactate dehydrogenase
ADP: Adenosine diphosphate
PDH: Pyruvate dehydrogenase
STAT3: Signal transducer and activator of transcription 3
FER: Feline encephalitis virus-related kinase
FerT: FER variant
CHCHD2: Coiled-coil-helix-coiled-coil-helix domain containing 2
PDK: Pyruvate dehydrogenase kinase
Uqcrfs1: Ubiquinol-cytochrome C reductase
COX5B: Cytochrome C oxidase subunit 5B
Ndufb8: NADH:ubiquinone oxidoreductase subunit B8
Uqcrc2: Ubiquinol-cytochrome C reductase core protein 2
PI3K: Phosphatidylinositol-4,5-bisphosphate 3-kinase
HIF-1α: Hypoxia-inducible factor 1 subunit alpha
NF-κB: Nuclear factor kappa B
ECL: Enhanced chemiluminescence
sh: Short hairpin
