# Supplementary figures and images for: SIRT6 enhances oxidative phosphorylation in breast cancer and promotes mammary tumorigenesis in mice
Source: Cancer Metab. 2021 Jan 22;9:6. doi: 10.1186/s40170-021-00240-1 (PMC7821730; doi:10.1186/s40170-021-00240-1)

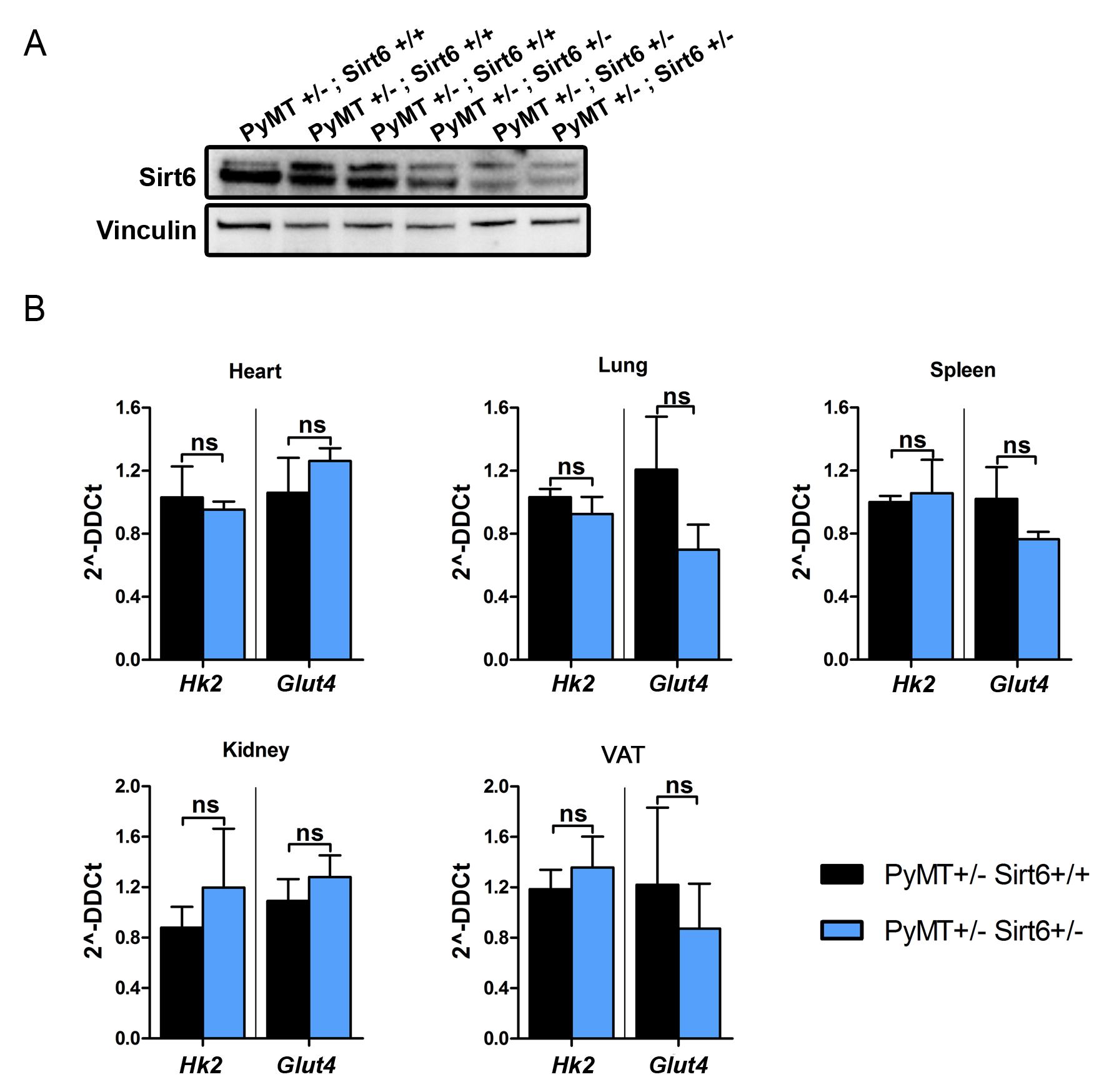

Supplement: Supplementary file 1 — Additional file 1: Fig. S1. Effect of Sirt6 heterozygous deletion on Sirt6, Glut4 and Hk2 expression in mouse mammary tumors and tissues. A, B, Mammary tumors and healthy tissues [heart, lung, spleen, kidney and visceral adipose tissue (VAT)] were isolated from twelve-week-old and from four-week-old MMTV-PyMT+/-; Sirt6+/+ (n = 3) and MMTV-PyMT+/-; Sirt6+/- (n = 3) mice, respectively, and used for protein lysate generation and for RNA extraction. In A, Sirt6 and vinculin levels were assessed by Western blot in mammary tumors. In B, Hk2 and Glut4 expression in bodily tissues was detected by QPCR. Data are presented as mean ± SD. ns: not statistically significant. [file 40170_2021_240_MOESM1_ESM.tif]

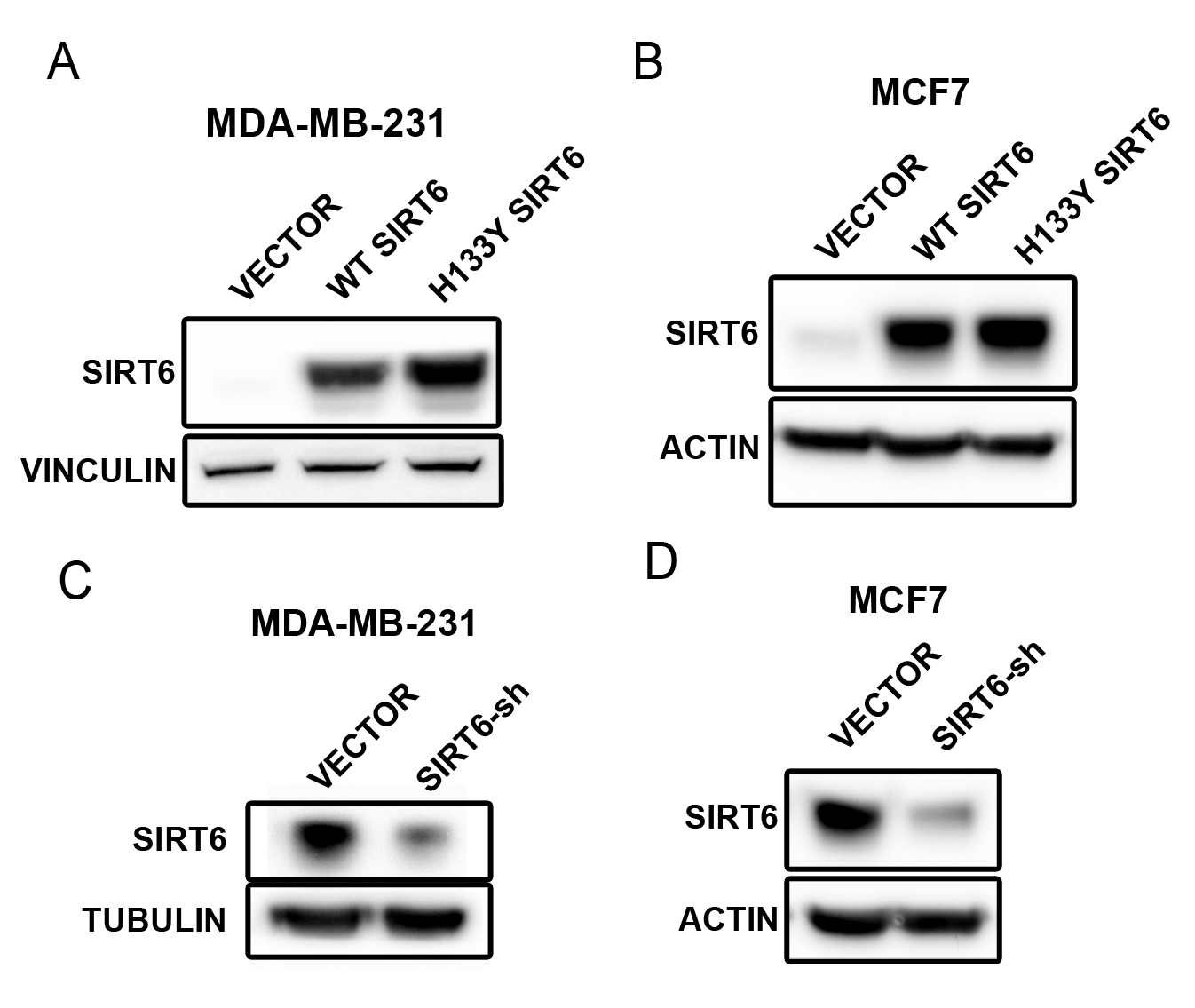

Supplement: Supplementary file 2 — Additional file 2: Fig. S2. SIRT6 silencing and overexpression in MDA-MB-231 and MCF7 cell lines. A, B, Western blot analysis of MDA-MB-231 (A) and MCF7 (B) cell lines overexpressing wild type (WT) or catalytically inactive (H133Y) SIRT6 with respect to control cells (VECTOR). C, D, Western blot analysis of MDA-MB-231 (C) and MCF7 (D) cell lines silenced for SIRT6 (SIRT6-sh) with respect to control cells (VECTOR). A-D, One representative experiment out of three is presented. [file 40170_2021_240_MOESM2_ESM.tif]

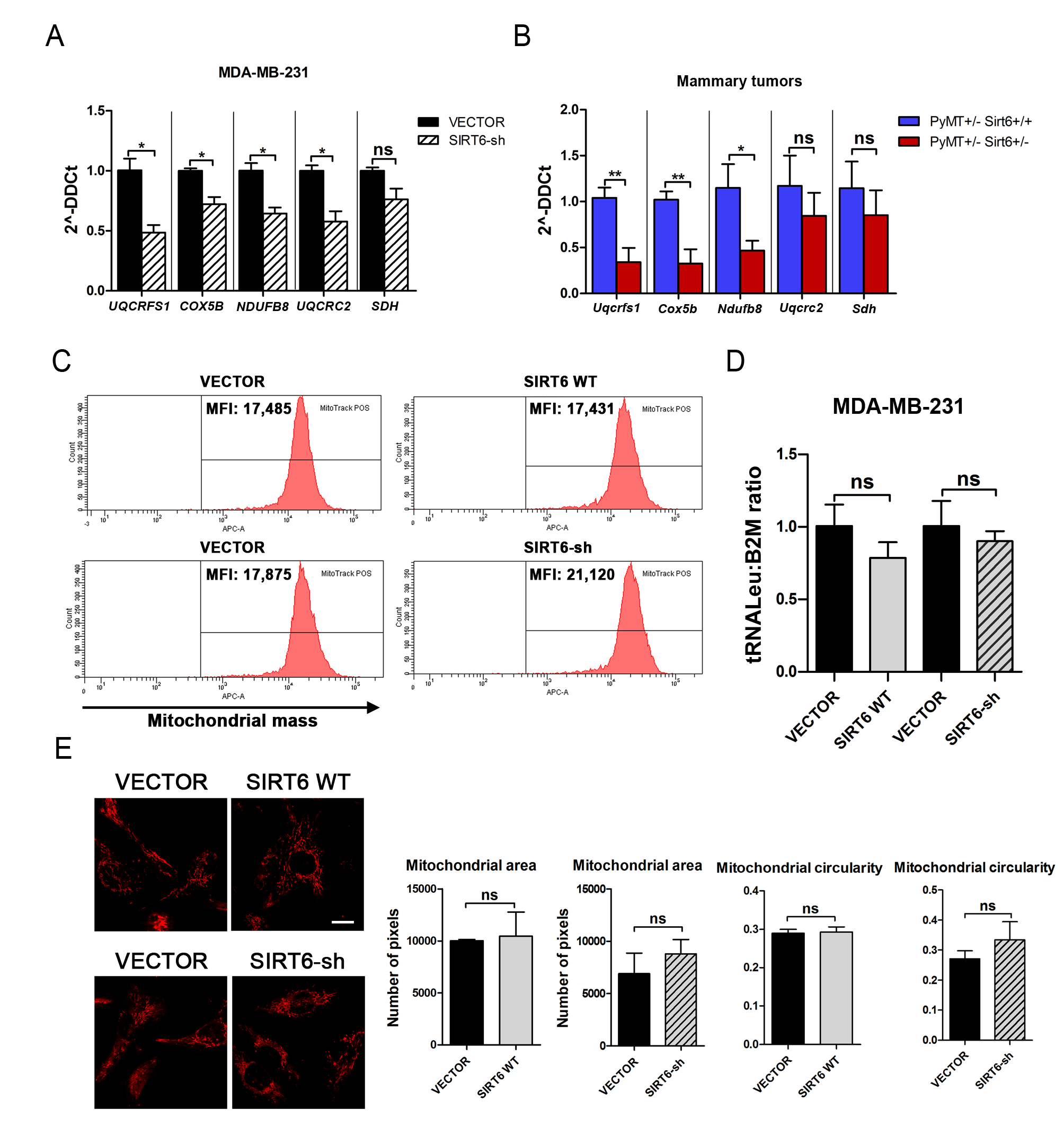

Supplement: Supplementary file 3 — Additional file 3: Fig. S3. Effect of SIRT6 deletion and overexpression on the expression of genes encoding for respiratory chain proteins and on mitochondrial mass and features. A, B, RNA was extracted from MDA-MB-231 cells engineered with a control vector (VECTOR) or a SIRT6-targeting shRNA (SIRT6-sh) (A) and from MMTV-PyMT+/-; Sirt6+/+ (n = 6) and MMTV-PyMT+/-; Sirt6+/- (n = 5) mice (B) and UQCRFS1, COX5B, NDUFB8, UQCRC2 and SDH expression was determined by QPCR. In A, data are presented as mean ± SD of three different experiments. *p<0.05, **p<0.01, ns: not statistically significant. C, D, MDA-MB-231 cells were engineered to overexpress WT SIRT6 (or a control vector) or to express a SIRT6-shRNA (or a control vector). Thereafter, cells were stained with Mitotracker deep red and analyzed by flow cytometry to detect their mitochondrial mass (C) or used for DNA extraction (D). In D, the amounts of DNA coding for tRNALeu and B2M were used to quantify mitochondrial and nuclear DNA, respectively, and their ratio was calculated. In C, one representative experiment out of three is presented. In D, data are presented as mean ± SD of three different experiments. ns: not statistically significant. (E) MDA-MB-231 cells that were engineered to overexpress WT SIRT6 (or a control vector) or a SIRT6-shRNA (or a control vector) were stained with Mitotracker deep red and analyzed by confocal microscopy, estimating mitochondrial area and circularity. Panel on the left shows one representative experiment out of three. In the histograms, data are presented as mean ± SD of at least three biological replicates. ns: not statistically significant. [file 40170_2021_240_MOESM3_ESM.tif]

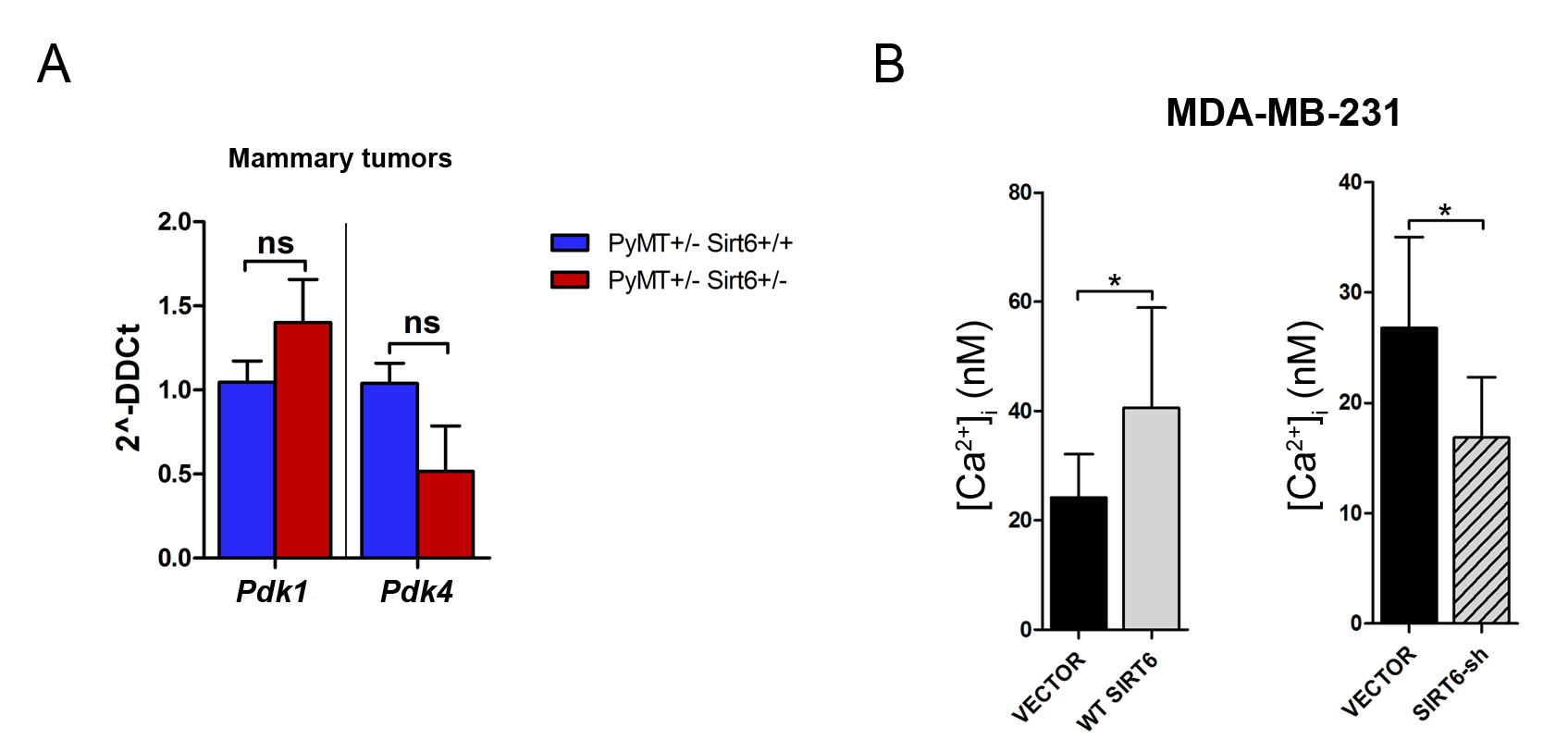

Supplement: Supplementary file 4 — Additional file 4: Fig. S4. SIRT6 levels do not affect Pdk1/4 expression but regulate intracellular calcium concentration in breast cancer cells. A, RNA was extracted from mammary tumors from twelve-week-old MMTV-PyMT+/-; Sirt6+/+ (n = 6) and MMTV-PyMT+/-; Sirt6+/- (n = 5) mice and Pdk1 and Pdk4 expression was determined by QPCR. B, MDA-MB-231 were engineered to overexpress WT SIRT6 (or a control vector) or to express a SIRT6-targeting shRNA (or a control vector). Thereafter, cells were used to measure intracellular calcium concentration. Data are presented as mean ± SD of three separate experiments. *p<0.05, ns: not statistically significant. [file 40170_2021_240_MOESM4_ESM.tif]

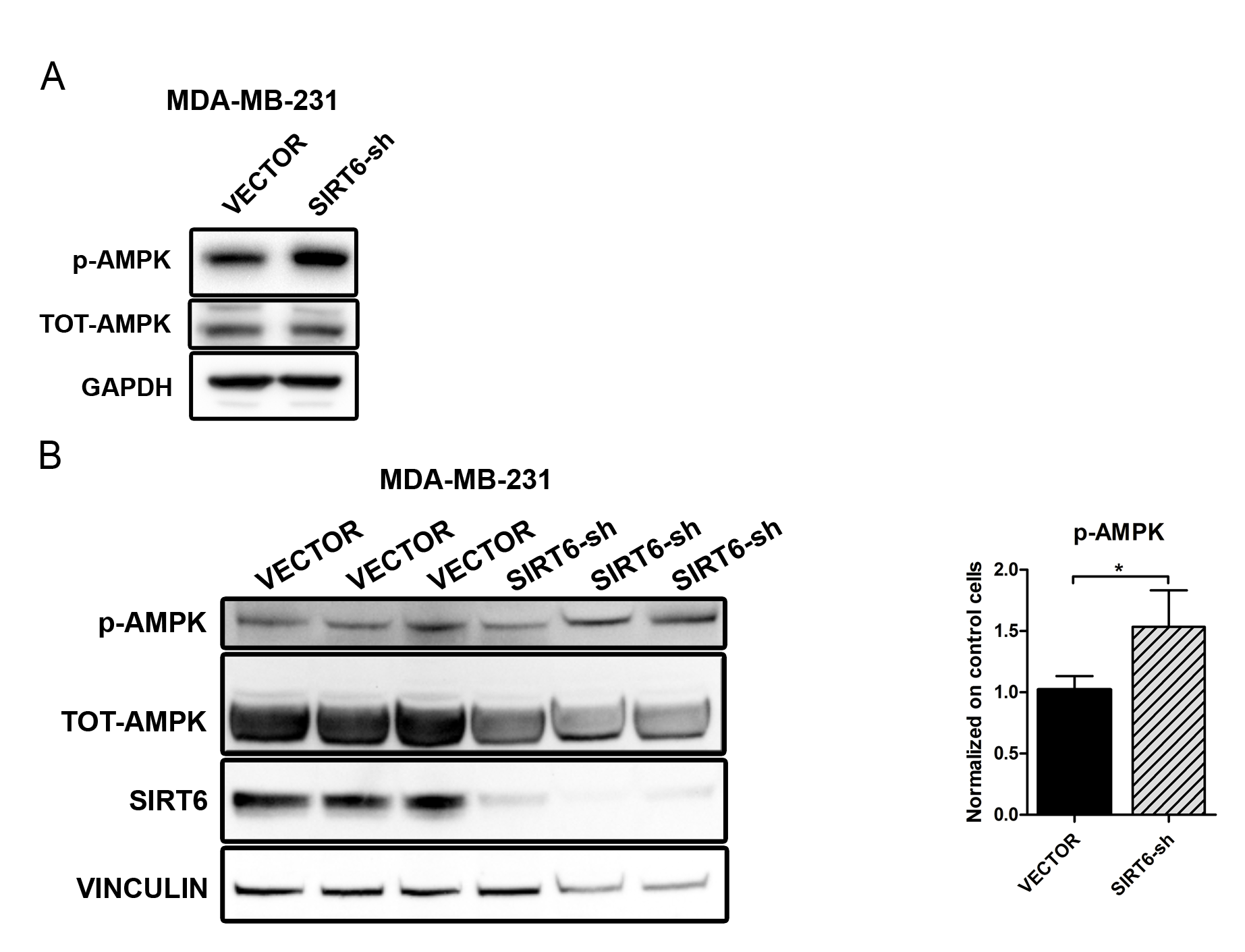

Supplement: Supplementary file 5 — Additional file 5: Fig. S5. SIRT6 silencing enhances AMPK phosphorylation in MDA-MB-231 xenografts. A, MDA-MB-231 cells were engineered to express a SIRT6-shRNA (or a control vector). Thereafter, cells were used for protein lysate generation and phosphorylated AMPK (Thr183, Thr172), total AMPK and GAPDH were detected by Western blot. One representative experiment out of three is presented. B, MDA-MB-231 BC cells transduced with either a SIRT6-shRNA or with a control vector were injected subcutaneously into both flanks of BALB/c athymic nude mice. Animals were sacrificed 50 days after cell inoculation; tumors were used for protein lysate generation and phosphorylated and total AMPK, SIRT6 and vinculin were detected by Western blot. In the right panel, the intensity of the phospho-AMPK bands was normalized to that of the total AMPK bands and the phospho-AMPK/total-AMPK ratio in tumors with silenced SIRT6 was compared to that detected in control tumors. Data are presented as mean ± SD. *p<0.05. [file 40170_2021_240_MOESM5_ESM.tif]

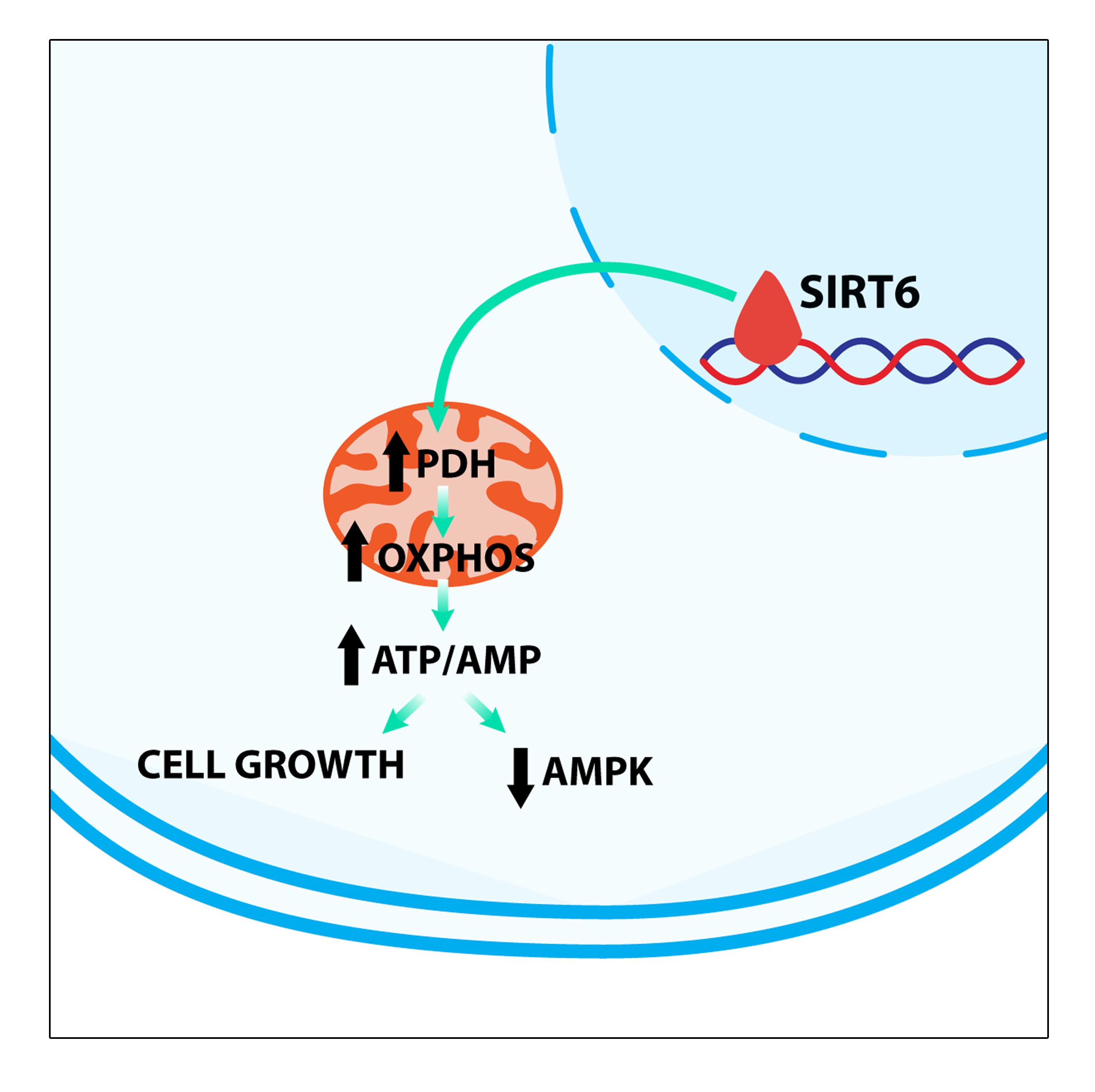

Supplement: Supplementary file 6 — Additional file 6: Fig. S6. Putative model for the metabolic, pro-oncogenic role of SIRT6 in breast tumorigenesis. SIRT6 increases expression and activity of PDH, as well as the levels of mitochondrial respiratory chain proteins. This results in increased OXPHOS and in a higher ATP/AMP ratio in BC cells. Ultimately, as a result of SIRT6 activity, AMPK activation is prevented and more ATP is available for cancer cells to grow. [file 40170_2021_240_MOESM6_ESM.tif]
