# Supplementary material for: SIRT6 enhances oxidative phosphorylation in breast cancer and promotes mammary tumorigenesis in mice
Source: Cancer Metab. 2021 Jan 22;9:6. doi: 10.1186/s40170-021-00240-1 (PMC7821730; doi:10.1186/s40170-021-00240-1)
Supplement: Supplementary file 7 — Additional file 7: Supplementary Table 1. QPCR primer list. [file 40170_2021_240_MOESM7_ESM.docx]

**Supplementary Table 1**. QPCR primer list

| **ACTIN** | FW | CGGGAAATCGTGCGTGACATTAAG |
| --- | --- | --- |
|  | REV | TGATCTCCTTCTGCATCCTGTCGG |
| **COX5B** | FW | TCTGTGAAGAGGACAATACC |
|  | REV | GCAATGGCTAGTCTTTACTG |
| **NDUFB8** | FW | ATGAGAGAGATCCATGGTATAG |
|  | REV | TATCATGAAAGCCAGGAAAC |
| **PDK1** | FW | ATGATGTCATTCCCACAATG |
|  | REV | AAGAGTGCTGATTGAGTAAC |
| **PDK4** | FW | GAATTATTGACCGCCTCTTTAG |
|  | REV | AGTAGAGATTCAGATCTCCTTG |
| **UQCRC2** | FW | GTGAGTCATCCTGTTCTAAAG |
|  | REV | CATTCTGTTCTCGGATTTCAC |
| **UQCRFS1** | FW | TCAATGTCCCTGCTTCTG |
|  | REV | TATCTAAAACTTCAAGGCGG |
| **SDHB** | FW | AGTTCTTATGCAGGCCTATC |
|  | REV | GGTATAGAGAGAATGGGTCC |
| **tRNALeu** | FW | CACCCAAGAACAGGGTTTGT |
|  | REV | TGGCCATGGGTATGTTGTTA |
| **B2M** | FW | TGCTGTCTCCATGTTTGATGTATCT |
|  | REV | TCTCTGCTCCCCACCTCTAAGT |
| **mouse *Actin*** | FW | GATGTATGAAGGCTTTGGTC |
|  | REV | TGTGCACTTTTATTGGTCTC |
| **mouse *Cox5b*** | FW | GGACCCATACAATATGCTAC |
|  | REV | CCACTATTCTCTTGTTGCTG |
| **mouse *Ndufb8*** | FW | CATGTGTAAACATCTCTTCGG |
|  | REV | TCCTCAGATATCATAGTGAACC |
| **mouse *Pdk1*** | FW | AGGATCTGACTGTGAAGATG |
|  | REV | TGGAAGTACTGTGCATAGAG |
| **mouse *Pdk4*** | FW | TTTGGCTGGTTTTGGTTATG |
|  | REV | TCCCATAACCTGACATAGAG |
| **mouse *Uqcrc2*** | FW | ATTCTGTCTTAAAGCAGGTG |
|  | REV | TCTCTAATTTCACCTCCACG |
| **mouse *Uqcrfs1*** | FW | ATATCCCTGAAGGGAAGAAC |
|  | REV | GATCTAATCATGCTGTGGG |
| **mouse *Sdhb*** | FW | TCTTGTAGAGAAGGCATCTG |
|  | REV | GACTAGATCCTTGATCACATAC |
| **mouse *Hk2*** | FW | CAAGCTACAGATCAAAGAGAAG |
|  | REV | CATGAGACCAAGAAACTCTC |
| **mouse *Glut4*** | FW | CAATGGTTGGGAAGGAAAAG |
|  | REV | AATGAGTATCTCATAGGAGGC |
